# Lactate-associated fibroblast–macrophage signaling in idiopathic pulmonary fibrosis: evidence boundaries and immunopharmacological opportunities

**DOI:** 10.3389/fphar.2026.1989965

**Published:** 2026-10-26

**Authors:** Yuntao Wu, Xinli Song, Bin Liu, Yuehua Han, Chen Zheng, Xiaoxu Zhao, Tianyu Zhu, Meng Yuan, Huihui Li, Yue Wang

**Affiliations:** 1 School of Integrated Traditional Chinese and Western Medicine, Tianjin University of Traditional Chinese Medicine, Tianjin, China; 2 Department of Infectious Diseases, Characteristic Medical Center of Chinese People’s Armed Police Force, Tianjin, China; 3 School of Medical Technology, Tianjin University of Traditional Chinese Medicine, Tianjin, China

**Keywords:** histone lactylation, idiopathic pulmonary fibrosis, immunometabolism, lactate, MCT4

## Abstract

Idiopathic pulmonary fibrosis (IPF) is characterized by recurrent alveolar epithelial injury, maladaptive repair, fibroblast activation, and extracellular-matrix accumulation. Lactate metabolism is increasingly implicated in communication between stromal and immune cells in fibrotic lung niches through monocarboxylate transporter (MCT)-mediated transport, hydroxycarboxylic acid receptor 1 (HCAR1/GPR81) sensing, extracellular acidification, and lysine lactylation. This narrative review evaluates the evidence for directional lactate-associated fibroblast–macrophage signaling, the limits of proposed reciprocal interactions, and their therapeutic implications. Human IPF observations, experimental pulmonary-fibrosis studies, and indirect evidence from other disease contexts are separated according to disease relevance and causal resolution. The strongest direct mechanistic evidence supports lactate released by activated myofibroblasts as a stimulus for macrophage histone lactylation and profibrotic transcription, whereas reciprocal macrophage-to-fibroblast lactate feedback remains unproven. Among the lactate-associated targets reviewed, fibroblast-associated MCT4 currently has the most developed preclinical evidence package, supported by disease-relevant observations, target perturbation, and antifibrotic activity in experimental models. However, no lactate-directed therapeutic strategy has yet demonstrated clinical efficacy in IPF. HCAR1/GPR81, lactate-production pathways, and lactylation-associated machinery remain mechanistically compelling but are at earlier stages of pharmacological validation. Overall, available evidence is more consistent with lactate functioning as a context-dependent amplifier or maintenance signal than with a demonstrated uniform initiating role in IPF. Lactate-directed interventions may therefore be considered adjunctive strategies alongside established antifibrotic therapy. Further translation will require cell- and disease-stage-specific causal validation, lesion-resolved target engagement, efficacy in established fibrosis, and a therapeutic window that preserves physiological lactate metabolism.

## Highlights

- Fibroblast-derived lactate promotes profibrotic macrophage programs.
- Reciprocal lactate-dependent feedback remains unproven in IPF.
- MCT4 has the most developed preclinical evidence among lactate targets.
- HCAR1/GPR81 and lactylation remain earlier-stage pharmacological targets.
- Translation requires cell-resolved target engagement in established fibrosis.

## 1 Introduction

Idiopathic pulmonary fibrosis (IPF) is a progressive fibrotic interstitial lung disease characterized by recurrent alveolar epithelial injury, maladaptive repair, persistent fibroblast and myofibroblast activation, and excessive extracellular matrix (ECM) deposition (Podolanczuk et al., 2023). Current pharmacological therapies can slow functional decline but do not reverse established structural fibrosis (Selvarajah et al., 2023). Pirfenidone and nintedanib remain established antifibrotic therapies, while the preferential phosphodiesterase 4B inhibitor nerandomilast has further broadened the contemporary therapeutic landscape following phase III clinical validation (Richeldi et al., 2025; Maher et al., 2025; Cruwys et al., 2024). Despite these advances, substantial unmet need remains for interventions that target mechanisms sustaining pathological remodeling and complement established antifibrotic therapy.

Fibrotic remodeling in IPF is accompanied by substantial metabolic and immune reprogramming within the injured lung microenvironment. Hypoxia, oxidative stress, inflammatory signaling, and ECM stiffening can reshape cellular metabolism and communication among epithelial, stromal, and immune compartments (Moss et al., 2022; Mutsaers et al., 2023). Macrophages occupy a central interface between injury clearance, tissue repair, and matrix remodeling, and their functional states are highly responsive to cytokine, metabolic, and matrix-derived cues (Mutsaers et al., 2023; Shenderov et al., 2021). These features provide a mechanistic basis for asking whether metabolites generated by activated stromal cells can function as signals that reinforce maladaptive immune–stromal communication during fibrotic progression.

Among these metabolic alterations, enhanced glycolysis and altered lactate homeostasis have emerged as potential mediators of intercellular communication in fibrotic lung tissue (Yan et al., 2024a). Activated fibroblasts and dysfunctional AEC2 cells show cell-specific alterations in lactate production, transport, or utilization (Ziehr et al., 2026; Newt et al., 2021). Accumulated lactate may regulate cellular behavior through monocarboxylate transporter (MCT)-mediated transmembrane exchange and hydroxycarboxylic acid receptor 1 (HCAR1/GPR81)-dependent sensing (Yang et al., 2023; Kobayashi et al., 2021). Lactate-associated extracellular acidification and histone lactylation provide additional mechanisms through which altered lactate metabolism may influence fibrotic responses (Cui et al., 2021; Kottmann et al., 2012). Most directly relevant to the present review, TGF-β1-stimulated pulmonary myofibroblasts release lactate, and myofibroblast-derived lactate can increase histone lactylation and profibrotic transcriptional programs in pulmonary macrophages (Cui et al., 2021). This provides direct mechanistic support for a stromal-to-immune metabolic axis, while the existence and functional importance of a broader reciprocal lactate-dependent circuit has not been established.

Previous reviews have summarized glycolytic reprogramming and lactate metabolism in IPF (Yan et al., 2024a), lactylation in pulmonary fibrosis (Li B. et al., 2026), and lactate–lactylation biology across fibrotic diseases (Liang et al., 2025). Recent reviews have also addressed the broader fibrotic microenvironment and immunometabolic reprogramming in pulmonary fibrosis. However, these broader syntheses do not fully resolve which lactate-centered immune–stromal interactions are directly supported in IPF-relevant systems, which remain inferential, and which lactate-associated nodes have sufficient target-level pharmacological validation for translational prioritization (Gao et al., 2025; Singh et al., 2026). The present review therefore adopts a narrower evidence-focused framework centered on lactate-associated fibroblast-to-macrophage signaling and candidate reciprocal immune–stromal interactions and separately evaluates disease provenance, causal resolution, and pharmacological maturity. Accordingly, rather than assuming a fully established fibroblast–macrophage circuit, this review uses fibroblast-to-macrophage lactate signaling as a mechanistic anchor and critically evaluates the evidence for putative reciprocal interactions within spatially organized fibrotic niches. Human IPF observations, mechanistic studies in experimental pulmonary fibrosis, and indirect evidence from other disease contexts are distinguished to separate disease relevance from mechanistic resolution. Candidate targets are further appraised according to causal validation, pharmacological tractability, target selectivity, target engagement, functional antifibrotic efficacy, systemic safety, and therapeutic-window considerations. The central objective is therefore not simply to establish whether lactate participates in pulmonary fibrosis, but to distinguish mechanistically plausible pathways from those with sufficient evidence for immunopharmacological translation.

## 2 Literature search strategy and evidence appraisal

Relevant English-language literature was identified through searches of PubMed and Web of Science Core Collection from database inception through 31 July 2026, supplemented by backward screening of reference lists from relevant primary studies and reviews. Search concepts combined “idiopathic pulmonary fibrosis” or “pulmonary fibrosis” with terms related to lactate biology, including “lactate,” “lactic acid,” “glycolysis,” and “lactylation,” together with cell- and pathway-specific terms including fibroblasts, macrophages, alveolar epithelial cells, MCT1/MCT4, HCAR1/GPR81, PFKFB3, LDHA, and PDK1. Searches were refined where necessary using pathway- or cell-specific terminology to identify studies addressing lactate production, transport, sensing, extracellular acidification, lactylation, and immune–stromal communication. Additional targeted searches used “lactate shuttle,” “lactate oxidation,” “LDHB,” “pyruvate,” and “TCA cycle” to identify studies addressing lactate utilization and oxidative metabolism.

Peer-reviewed original studies using human IPF tissues, patient-derived cells, *ex vivo* human models, animal models, or disease-relevant mechanistic cellular systems were prioritized. Particular emphasis was placed on studies that directly assessed lactate production, transport, sensing, lactylation, or functional consequences relevant to pulmonary fibrosis. Studies in which lactate was measured only as a nonspecific metabolic endpoint, without mechanistic linkage to lactate production, transport, sensing, acidification, or lactylation, were not used to support lactate-specific causal claims. Studies addressing general glycolysis without a clear connection to lactate biology were used only when necessary to establish mechanistic context. Evidence from non-IPF disease settings was included only when it informed a specific mechanistic question not adequately resolved by IPF or pulmonary-fibrosis studies and was explicitly treated as indirect evidence. Reviews were used primarily to contextualize established concepts and identify additional primary studies rather than as the principal basis for mechanistic conclusions.

For primary mechanistic studies, additional study-level variables were extracted where reported, including the biological compartment in which lactate was measured or manipulated, the analytical method, absolute or relative lactate abundance, exogenous lactate concentration and exposure duration, extracellular pH and pH-control conditions, relevant cell type, donor–recipient configuration, lactate-specific perturbation, downstream mechanistic readout, and the principal limitation affecting interpretation. Particular attention was paid to whether observed effects could be attributed specifically to lactate rather than to co-varying extracellular acidification, broader glycolytic reprogramming, or other soluble components present in conditioned media or biological fluids. These study-level variables and the rationale for evidence-tier assignment are summarized in Supplementary Table S1.

Evidence was classified into three source-based tiers according to disease context and experimental design; these tiers describe evidence provenance rather than a hierarchical ranking of overall evidentiary strength. Tier designation therefore reflects disease context and evidence provenance, whereas causal resolution is evaluated separately. Tier A comprised evidence directly derived from human IPF tissues, patient-derived cells, or *ex vivo* human material, irrespective of whether the study was observational or interventional. Tier B comprised mechanistic evidence from experimental pulmonary-fibrosis models or non-human pulmonary-fibrosis-relevant cellular systems in which lactate abundance, production, transport, sensing, extracellular acidification, or downstream lactylation was directly perturbed. Tier C comprised indirect or hypothesis-generating evidence from non-IPF disease contexts, general lactate biology, or mechanistic studies not yet validated in pulmonary fibrosis. Accordingly, Tier A provides the greatest direct relevance to human IPF but does not necessarily establish causality, whereas Tier B may provide greater mechanistic resolution through controlled experimental perturbation despite lower direct human disease relevance.

Pharmacological appraisal considered human disease relevance, causal target validation, availability and selectivity of pharmacological tools, evidence of target engagement, efficacy when intervention was initiated after fibrosis establishment rather than exclusively in preventive paradigms, functional antifibrotic outcomes, and potential systemic safety constraints. Targets were considered more translationally mature when supported by convergent evidence of disease relevance, target-specific perturbation, pharmacological target engagement, efficacy in established fibrosis, and functional antifibrotic benefit, whereas targets supported primarily by expression changes or nonselective pharmacological interventions were considered less mature. This review was conducted as a narrative evidence synthesis rather than a systematic review or meta-analysis; therefore, no formal risk-of-bias assessment or quantitative evidence synthesis was performed. Accordingly, the A/B/C categories should not be interpreted as formal levels of evidence or as numerical rankings of therapeutic priority. The evidence tiers and translational priorities should consequently be interpreted as a structured qualitative appraisal rather than formal evidence grading or clinical recommendations.

Using this source-based evidence-appraisal framework, Figure 1 presents an integrative working model that distinguishes comparatively well-supported lactate-associated mechanisms from proposed or incompletely connected interactions. A study-level evidence map of lactate-associated mechanisms, immune–stromal interactions, and translational relevance is provided in Table 1.

**FIGURE 1:**
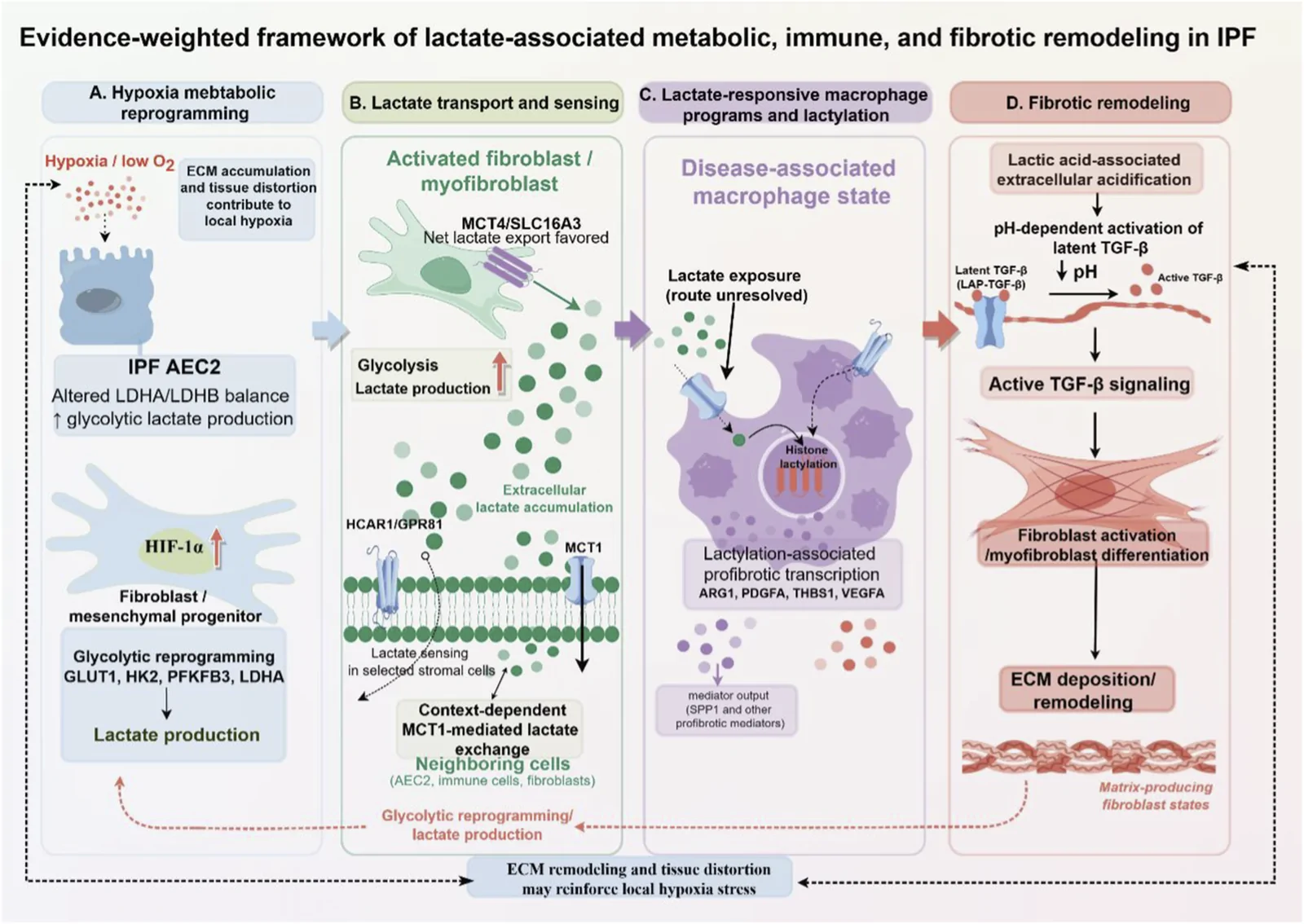
Evidence-weighted framework of lactate-associated metabolic, immune, and fibrotic remodeling in idiopathic pulmonary fibrosis. Hypoxic or pseudohypoxic HIF activation can promote glycolytic reprogramming and altered lactate metabolism in fibrotic lung cells. Activated fibroblasts and myofibroblasts currently provide the strongest evidence for increased extracellular lactate production and release, with MCT4/*SLC16A3* preferentially supporting net lactate export under highly glycolytic or hypoxic conditions. MCT1/*SLC16A1* mediates context- and gradient-dependent bidirectional transport, whereas HCAR1/GPR81 provides a distinct mechanism of extracellular lactate sensing in selected stromal populations. Experimental pulmonary-fibrosis studies support a fibroblast-to-macrophage lactate axis in which increased lactate availability promotes macrophage histone lactylation and profibrotic transcriptional outputs. Reciprocal macrophage-to-stromal profibrotic signaling is biologically plausible but has not been connected to fibroblast-derived lactate within a closed causal circuit. Lactic-acid-associated extracellular acidification can additionally facilitate pH-dependent activation of latent TGF-β and reinforce fibroblast activation and matrix remodeling. Human IPF-derived AEC2 cells show disease-associated lactate metabolic dysregulation, although their quantitative contribution to the extracellular lactate pool remains unresolved. ECM remodeling and tissue distortion are shown as potential feedbacks to local hypoxia. Solid arrows indicate comparatively well-supported mechanistic relationships across the evidence sources reviewed, whereas dashed arrows denote proposed or incompletely connected relationships; neither should be interpreted as evidence of complete causal validation in human IPF.

**TABLE 1:** Evidence map of lactate-associated mechanisms, immune–stromal interactions, and translational relevance in idiopathic pulmonary fibrosis.

Mechanistic question/pathway	Representative study/system	Relevant cell type(s)	Lactate-related intervention or readout	Main mechanistic finding	Evidence provenance	Causal resolution	Key limitation
Lactic acid–TGF-β coupling	Kottmann et al.; human IPF lung tissue and primary human lung fibroblasts	Fibroblasts	Lactic acid/LDH5 measurement; extracellular pH manipulation; TGF-β activation	Human IPF lungs show increased lactic acid and LDH5; lactic-acid-associated extracellular acidification promotes activation of latent TGF-β and myofibroblast differentiation	A	Human observation + cell perturbation	Does not establish that lactate anion and extracellular acidification are mechanistically interchangeable
AEC2 lactate metabolic dysregulation	Newton et al.; primary AEC2 isolated from IPF explants	AEC2	LDHA/LDHB profile; lactate production; LDHA siRNA	IPF-derived AEC2 show impaired oxidative metabolism, increased glycolytic lactate production, and altered LDHA/LDHB balance; LDHA reduction partially restores oxidative metabolism	A	Patient-derived cell perturbation	Does not establish the quantitative contribution of AEC2-derived lactate to the extracellular lactate pool *in vivo*
HIF–lactate–HCAR1/GPR81 signaling	Yang et al.; IPF-derived mesenchymal progenitor cells	Mesenchymal progenitor cells	Hypoxia/HIF-1α; LDHA/lactate; GPR81 expression and knockdown; promoter occupancy	HIF-1α-dependent regulation of GPR81 contributes to lactate-associated self-renewal, migration, and fibrogenicity in IPF-derived stromal cells	A	Genetic perturbation + promoter occupancy	Evidence is concentrated in a selected IPF stromal population and does not establish universal HCAR1 dependence across IPF cell types
Persistent-hypoxia-associated lactate production	Patient-derived control and IPF fibroblast study	Fibroblasts	Extracellular lactate; LDHA/LDHB regulation; GPR81	Persistent hypoxia increases extracellular lactate and alters LDH isoenzyme regulation; lactate/GPR81 responses vary according to fibroblast disease state	A	Patient-derived functional study	*Ex vivo* system; lesion-level lactate flux and receptor dependence remain unresolved
MCT4-dependent lactate export and paracrine signaling	2026 patient-derived IPF fibroblast study	IPF fibroblast donor → nonfibrotic fibroblast recipient	MCT4 expression, silencing or pharmacological inhibition; extracellular lactate; conditioned medium	Hypoxia increases MCT4-dependent extracellular lactate accumulation; donor-cell MCT4 perturbation attenuates profibrotic paracrine signaling	A	Donor-cell perturbation	Recipient system does not reproduce the complete multicellular IPF niche; direct *in situ* lactate transfer remains unproven
Fibroblast-to-macrophage lactate signaling	Cui et al.; myofibroblast-conditioned medium, fibrotic BALF and experimental pulmonary fibrosis	Myofibroblast → macrophage	Conditioned medium; exogenous lactate; histone lactylation; promoter ChIP; p300 knockdown	Increased lactate availability promotes macrophage histone lactylation and expression of profibrotic mediators including Arg1, Spp1, pdgfa, Thbs1 and vegfa	B	Donor–recipient + downstream perturbation	Conditioned medium contains other soluble mediators; matched human IPF donor–recipient causality and closed feedback are not demonstrated
MCT-dependent lactate transport, utilization, and fibroblast metabolic reprogramming	Ziehr et al., 2026; fibroblast systems and experimental pulmonary fibrosis	Fibroblasts/fibrotic lung	Genetic and pharmacological MCT1/MCT4 inhibition; MCT4-directed VB253; 2 mM [U-^13^C_3_]-lactate tracing	MCT perturbation reduces TGF-β1-induced myofibroblast differentiation and experimental fibrosis; isotope tracing demonstrates incorporation of extracellular lactate-derived carbon into intracellular pyruvate and TCA-cycle intermediates	B	Genetic/pharmacological perturbation + *in vivo* therapeutic efficacy	Preclinical evidence; does not establish donor-specific fibroblast-to-macrophage lactate transfer or oxidative utilization in human IPF; independent validation of compound selectivity, PK, and safety remains required
Macrophage-to-fibroblast metabolic regulation	Xu et al.; LPS-driven experimental pulmonary fibrosis	Macrophage → fibroblast	Macrophage JNK/TNF-α and fibroblast TNFR1/PFKFB3 perturbation; glycolysis/lactate measurements	Macrophage inflammatory signaling increases fibroblast PFKFB3-dependent glycolysis and lactate production	B	Immune-to-stromal pathway perturbation	Inflammation-dominant LPS model; does not demonstrate that the macrophage response was initiated by fibroblast-derived lactate
Non-lactate-specific myeloid–mesenchymal metabolic crosstalk	Yadav et al.; experimental pulmonary fibrosis + human IPF precision-cut lung slices	Myeloid cells → fibroblasts	ARG1/ornithine pathway perturbation	Myeloid ARG1-dependent ornithine metabolism can support fibroblast proline/collagen synthesis, demonstrating that reciprocal immune–mesenchymal metabolic circuits are biologically feasible	B	Cell-specific perturbation + *ex vivo* human validation	Pathway is not lactate-dependent; ARG1-expressing cells differ between murine and human disease
Epithelial MCT1-dependent lactate handling	Feng et al.; LPS-driven fibrosis model	Alveolar epithelial cells	MCT1 expression/manipulation; lactate accumulation; epithelial/mesenchymal markers	Reduced epithelial MCT1 is associated with disturbed lactate handling and EMT-like epithelial plasticity	B	Genetic/functional perturbation	Inflammation-dominant model; should not be extrapolated to complete EMT or human IPF epithelial biology
Akt2–PDK1-dependent pyruvate partitioning	Sun et al.; experimental pulmonary fibrosis	Fibroblasts/fibrotic lung	Akt2–PDK1 pathway perturbation; lactate accumulation	Altered pyruvate handling promotes lactate accumulation and fibrotic remodeling	B	Pathway perturbation	Selective PDK1-directed pharmacological validation in established pulmonary fibrosis remains limited
Lactate-associated epithelial ER stress	Sun et al.; bleomycin fibrosis and alveolar epithelial-like cell systems	Alveolar epithelial-like cells	Exogenous lactate; ATF4–CHOP-associated ER-stress signaling; ER-stress inhibition	Lactate-associated ER stress promotes epithelial apoptosis; pharmacological ER-stress attenuation reduces epithelial injury and experimental fibrosis	B	Pharmacological rescue	Key cellular experiments were not performed in primary human IPF AEC2 cells
Site-specific macrophage histone lactylation	Recent non-IPF toxicant-driven pulmonary-fibrosis model	Macrophages	Lactate production; H3K18la; locus-specific chromatin assessment; TGF-β1 regulation	Provides residue- and locus-specific evidence linking macrophage lactate metabolism to profibrotic transcriptional/secretory outputs	B	Site/locus-specific mechanistic evidence + pathway perturbation	Etiologically distinct from IPF; direct relevance to human IPF macrophages remains unvalidated
AARS1/AARS2-mediated lysine lactylation	Li et al.; general mechanistic systems	Multiple cell types	L-lactate sensing; ATP-dependent lysine lactylation	AARS1/AARS2 can function as intracellular L-lactate sensors and broad lysine lactyltransferases	C	Biochemical/mechanistic perturbation	No direct validation in pulmonary macrophage histone lactylation, fibroblast–macrophage crosstalk, or human IPF
MCT1 clinical pharmacological tractability	Halford et al.; first-in-human phase I oncology study of AZD3965	Systemic/tumor context	Oral selective MCT1 inhibition; clinical dose escalation and safety assessment	Human systemic MCT1 inhibition is pharmacologically achievable; 10 mg twice daily selected as RP2D	C	Human pharmacology without IPF disease validation	Oncology context; no IPF efficacy or IPF-specific target engagement; systemic toxicities limit direct extrapolation
Direct extracellular lactate elimination	Xu et al., 2025; CeO_2_@PLP in bleomycin pulmonary fibrosis	Fibrotic lung/macrophage–fibroblast compartments	LOx-mediated lactate degradation; CeO_2_@PLP; tissue lactate and fibrosis readouts	A lactate-scavenging nanoplatform reduced lactate accumulation and improved macrophage-/fibroblast-associated fibrotic outcomes	B	*In vivo* proof of concept	Multicomponent platform combines LOx, CeO_2_, targeting, and pirfenidone; total efficacy cannot be attributed uniquely to lactate removal; no human IPF validation

Evidence provenance indicates disease context and source of evidence rather than overall evidentiary strength: A, human IPF-derived evidence; B, experimentally perturbed pulmonary-fibrosis evidence; C, indirect or hypothesis-generating evidence. Causal resolution is evaluated separately.

## 3 Sources, transport, and sensing of lactate in IPF

### 3.1 Hypoxia, HIF-1α activation, and glycolytic reprogramming

The distorted architecture of fibrotic IPF lung, including alveolar destruction, extracellular-matrix expansion, and microvascular remodeling, may restrict local oxygen delivery and diffusion (Moss et al., 2022; Bian et al., 2023). More direct evidence of hypoxia-associated signaling comes from human IPF tissue, in which HIF-1α, HIF-2α, and carbonic anhydrase IX (CAIX) are enriched around the periphery of fibroblastic foci and spatially overlap with IPF-associated mesenchymal progenitor cells (MPCs) (Yang et al., 2023). These observations support the presence of spatially restricted hypoxia-associated signaling within fibrotic lesions. Importantly, HIF pathway activation should not be treated as a direct surrogate for tissue hypoxia. Human lung-fibrosis studies also demonstrate normoxic, or “pseudohypoxic,” HIF activation associated with oxidative stress, reduced activity of factor inhibiting HIF (FIH), and altered collagen remodeling (Brereton et al., 2022). Thus, oxygen limitation and oxygen-independent HIF activation may coexist within fibrotic niches and should be mechanistically distinguished.

Under oxygen-replete conditions, HIF-1α is hydroxylated and targeted for VHL-dependent degradation, whereas oxygen limitation stabilizes HIF-1α and promotes transcriptional programs that favor glycolysis. Relevant targets include GLUT1, HK2, PFKFB3, LDHA, and PDK1, collectively increasing glucose utilization and favoring pyruvate diversion toward lactate production (Kierans and Taylor, 2021; Taylor and Scholz, 2022). Human IPF tissue and complementary fibroblast–matrix and experimental fibrosis models further implicate HIF-1α-associated signaling in profibrotic matrix remodeling (Epstein Shochet et al., 2021; Xie et al., 2015; Hu et al., 2020; Cho et al., 2017).

Several complementary lines of IPF-relevant evidence provide mechanistic links between hypoxic/HIF-associated signaling and lactate-related fibrotic phenotypes. Studies of MPCs derived from human IPF lungs show that exposure to 2% O_2_ increases HIF-1α abundance, LDHA expression, lactate production/release, and HCAR1/GPR81 expression. Quantitatively, lactate abundance in IPF-derived MPCs was approximately 31% higher than in control MPCs under normoxia, whereas the difference increased to approximately 97% under hypoxia; within IPF MPCs, hypoxia increased lactate by approximately 65% relative to normoxia (Yang et al., 2023). HIF-1α knockdown reduces hypoxia-induced HCAR1/GPR81 expression, while chromatin immunoprecipitation demonstrates HIF-1α occupancy at the GPR81 promoter. Functionally, exogenous lactate tested across 0–20 mM promoted IPF MPC self-renewal, whereas GPR81 knockdown suppressed hypoxia-induced self-renewal and attenuated fibrogenicity in experimental models (Yang et al., 2023). Complementary studies in primary lung fibroblasts further demonstrate disease-state-dependent regulation of this pathway. Under persistent hypoxia (1% O_2_ for 5 days), IPF-derived fibroblasts show increased extracellular lactate production associated with a higher LDHA/LDHB ratio, driven in part by FoxM1-dependent LDHA regulation. Hypoxia also increases GPR81 expression in both control and IPF fibroblasts; however, the phenotypic response to exogenous lactate and GPR81 perturbation differs between control and IPF fibroblasts, indicating that lactate responsiveness is not uniform across fibroblast states (Nho et al., 2023). Exogenous lactate was examined across 0.5–5 mM, with 5 mM used in key differentiation experiments. Importantly, selected experiments included adjustment of extracellular pH to approximately 7.3, supporting a component of the fibroblast response that was not attributable solely to extracellular acidification. Pharmacological interpretation of 3-hydroxybutyrate used as a putative GPR81 antagonist should nevertheless be treated cautiously because this compound is not a validated selective HCAR1/GPR81 antagonist; the accompanying GPR81 knockdown therefore provides stronger receptor-specific evidence. CCT6A studies provide complementary evidence for epithelial regulation of HIF-1α-dependent lactate production. Human fibrotic-lung tissue shows increased CCT6A expression in AEC2 cells, whereas mechanistic experiments performed primarily in AT2-like epithelial cells demonstrate that CCT6A gain of function increases VHL abundance and promotes VHL-dependent HIF-1α ubiquitination and degradation, thereby reducing glycolytic activity and extracellular lactate production (Yan et al., 2024b). *In vivo*, *Cct6a* overexpression attenuates bleomycin-induced pulmonary fibrosis and reduces circulating lactate, whereas *Cct6a* loss of function aggravates fibrotic injury (Yan et al., 2024b). The apparent dissociation between disease-associated CCT6A upregulation and its antifibrotic gain-of-function phenotype cautions against interpreting increased CCT6A abundance itself as evidence of a profibrotic role. Together, these studies position HIF-1α-associated signaling as an important regulator connecting cellular stress to lactate-associated profibrotic phenotypes. However, they do not establish that oxygen limitation itself is the dominant cause of HIF-1α activation, nor that the HIF-1α–lactate axis has equivalent causal importance across epithelial, mesenchymal, and immune compartments or across disease stages.

Interpretation of HIF-1α requires distinction among transcript abundance, protein stabilization, transcriptional activity, and downstream metabolic flux. *HIF1A* mRNA abundance alone does not establish stabilization of the oxygen-sensitive HIF-1α protein, while increased HIF-1α immunoreactivity does not by itself demonstrate enhanced glycolytic or lactate flux. Strong causal attribution therefore requires coupling cell-specific HIF perturbation with direct metabolic measurements, such as stable-isotope tracing, extracellular flux analysis, or spatially resolved lactate measurements. These approaches are also necessary to distinguish lactate-dependent effects from broader HIF-1α functions in proliferation, survival, vascular adaptation, and cellular stress responses.

### 3.2 Cellular sources and regulation of lactate homeostasis

Activated fibroblasts and myofibroblasts currently provide the strongest evidence for a stromal source of extracellular lactate in pulmonary fibrosis, with support from both experimental fibrosis systems and patient-derived IPF fibroblasts. TGF-β1, persistent hypoxia, and inflammatory signaling can enhance fibroblast glycolysis and shift pyruvate metabolism toward lactate production (Cui et al., 2021; Nho et al., 2023; Xu et al., 2022). Early studies provided quantitative evidence of altered lactate-associated metabolism in human IPF and primary lung fibroblasts. Kottmann et al. measured lactic acid in whole-lung homogenates from patients with IPF and non-fibrotic controls (n = 6 per group) using high-resolution ^1H-PASS nuclear magnetic resonance spectroscopy and reported a significant increase in IPF lung tissue; this difference was subsequently summarized as approximately threefold in an independent IPF AEC2 study (Newt et al., 2021; Kottmann et al., 2012). In the same study, extracellular lactate was measured in supernatants from primary human lung fibroblasts treated with 5 ng/mL TGF-β (n = 3 per condition), also using ^1H-PASS NMR. TGF-β-induced lactate accumulation was accompanied by an approximately 0.6-unit decrease in culture-medium pH from a baseline of 7.8 over 72 h (Kottmann et al., 2012). These measurements provide direct quantitative support for increased lactate-associated metabolism but derive from bulk lung homogenates or cell-culture supernatants rather than spatially resolved human IPF lesions. More recent studies of patient-derived IPF fibroblasts demonstrate increased extracellular lactate under persistent hypoxia, associated with altered LDHA/LDHB regulation and enhanced MCT4 expression; MCT4 silencing or pharmacological inhibition reduces extracellular lactate accumulation (Nho et al., 2023; Nho et al., 2026). Together, these findings provide direct evidence that activated fibroblast populations can contribute to the extracellular lactate pool, although their quantitative contribution within human fibrotic lesions remains unresolved (Xie et al., 2015; Hu et al., 2020; Cho et al., 2017; Tang et al., 2021; O’Leary et al., 2020; Bueno et al., 2020).

AEC2 cells also exhibit dysregulated lactate metabolism in IPF. Primary AEC2 cells isolated from IPF lung explants show reduced oxidative metabolic capacity, increased glycolytic lactate production, and LDH isoenzyme profiles with a higher LDHA/LDHB ratio than non-fibrotic control AEC2 cells (Newt et al., 2021). Importantly, siRNA-mediated reduction of LDHA in IPF-derived AEC2 cells shifts LDH isoenzyme composition and partially restores a more oxidative bioenergetic profile, providing functional support for a cell-intrinsic contribution of LDH dysregulation (Newt et al., 2021). However, these *ex vivo* findings do not establish that AEC2 cells are a quantitatively dominant source of extracellular lactate *in vivo*. Their contribution to the lactate pool within fibrotic lesions, and how this contribution varies with disease stage or epithelial cell state, remain unresolved.

Immune cells may also regulate lactate homeostasis indirectly rather than acting primarily as demonstrated sources of extracellular lactate. In an LPS-driven model of inflammation-associated pulmonary fibrosis, LPS-stimulated macrophages activate JNK signaling and release TNF-α, which subsequently promotes PFKFB3-dependent aerobic glycolysis and lactate production in lung fibroblasts (Xu et al., 2022). Pharmacological or genetic interruption of the macrophage JNK–TNF-α/fibroblast TNFR1–PFKFB3 pathway reduces fibroblast glycolysis and attenuates fibrosis in this experimental setting (Xu et al., 2022). These findings demonstrate an immune-to-stromal metabolic regulatory pathway, but because the evidence derives from an inflammation-dominant LPS model rather than human IPF, it should not be interpreted as an established macrophage-to-fibroblast mechanism in IPF. Macrophages should therefore be considered potential regulators of stromal lactate production in this context rather than demonstrated major sources of the extracellular lactate pool (Xie et al., 2015).

Elevated lactate should be interpreted at both concentration and flux levels. Tissue or BALF lactate concentrations reflect the net balance among cellular production, uptake and oxidation, transmembrane transport, and vascular clearance, while local pH can further influence proton-coupled MCT transport and compartmental distribution (Kobayashi et al., 2021). Static endpoint measurements therefore cannot identify the dominant cellular source or distinguish increased production from reduced utilization or clearance. Stable-isotope tracing, cell-type-resolved metabolic measurements, and spatial metabolomic approaches will be required to resolve lactate exchange within fibrotic niches. Because metabolite measurements are also sensitive to sampling and tissue-processing conditions, comparisons across experimental systems should be interpreted cautiously (Epstein Shochet et al., 2021; Wel et al., 2022; Morland et al., 2015).

### 3.3 MCT-mediated transport and HCAR1/GPR81 sensing

Lactate does not freely diffuse across the plasma membrane to an extent sufficient to account for regulated cellular exchange, and its transmembrane movement is mediated primarily by proton-coupled monocarboxylate transporters. MCT4 (*SLC16A3*) is commonly enriched in highly glycolytic or hypoxic cells and, under these conditions, generally supports net lactate export. MCT1 (*SLC16A1*), by contrast, can support lactate uptake or export depending on cellular identity, metabolic state, and the prevailing lactate and proton gradients (Ziehr et al., 2026). Accordingly, MCT1 and MCT4 function should not be inferred solely from expression levels or simplified labels of “importer” and “exporter,” but interpreted in the context of intracellular and extracellular lactate concentrations, pH, and cellular metabolic demand (Ziehr et al., 2026; Xie et al., 2015; Zhang et al., 2024).

Recent studies provide comparatively strong causal evidence that lactate transport is required for fibroblast metabolic reprogramming and myofibroblast differentiation. Genetic and pharmacological inhibition of MCT1 or MCT4 reduces TGF-β1-induced myofibroblast differentiation and attenuates bleomycin-induced pulmonary fibrosis, with MCT4-directed inhibition showing greater antifibrotic activity than MCT1-directed inhibition within the same experimental framework (Ziehr et al., 2026). Stable-isotope tracing using 2 mM [U-^13^C_3_]-lactate further demonstrated incorporation of extracellular lactate-derived carbon into intracellular pyruvate and TCA-cycle intermediates (Ziehr et al., 2026). The implications of these findings for lactate utilization and shuttle logic are discussed below. This comparative advantage should therefore be interpreted as model-specific rather than as evidence that MCT4 inhibition will uniformly outperform MCT1-directed approaches across disease contexts. The MCT4 inhibitor VB253 further attenuates fibrosis in both young and aged mice, providing pharmacological proof of concept for transport-directed intervention (Ziehr et al., 2026). Complementary studies using patient-derived IPF fibroblasts show that hypoxia increases MCT4 expression and extracellular lactate accumulation, whereas MCT4 silencing or pharmacological inhibition reduces extracellular lactate levels (Nho et al., 2026). Conditioned medium from hypoxic IPF fibroblasts increases α-SMA expression in recipient nonfibrotic lung fibroblasts, and this effect is attenuated by MCT4 inhibition in donor IPF fibroblasts or GPR81-directed intervention in recipient cells (Nho et al., 2023; Nho et al., 2026). Together, these findings provide direct experimental support for a donor-cell MCT4-dependent lactate export–recipient-cell lactate-sensing axis in fibroblast-to-fibroblast profibrotic signaling under hypoxic conditions, although direct demonstration of such lactate transfer within human IPF lesions remains lacking (Xie et al., 2015; Zhang et al., 2024).

The consequences of MCT manipulation are nevertheless cell- and context-dependent. In an LPS-driven mouse model of inflammation-associated pulmonary fibrosis and complementary MLE-12 epithelial-cell experiments, reduced epithelial MCT1 expression was associated with impaired lactate handling, extracellular lactate accumulation, and EMT-like phenotypic changes, whereas MCT1 restoration attenuated these effects (Feng et al., 2024). Because these findings derive from an inflammation-dominant mouse model rather than human IPF, they should not be interpreted as evidence that epithelial MCT1 inhibition is uniformly detrimental in IPF. Instead, they illustrate why the therapeutic consequences of MCT blockade depend on cellular identity, metabolic directionality, and disease context.

HCAR1/GPR81 provides a receptor-mediated mechanism of extracellular lactate sensing that is mechanistically distinct from transmembrane lactate transport (Certo et al., 2022). In MPCs derived from human IPF lungs, hypoxia increases HCAR1/GPR81 expression through HIF-1α-dependent transcriptional regulation, while exogenous lactate promotes self-renewal and GPR81 knockdown attenuates hypoxia-induced profibrotic behavior and fibrogenicity (Yang et al., 2023). Complementary fibroblast studies indicate substantial disease-state dependence: persistent hypoxia increases GPR81 expression in both nonfibrotic and IPF-derived fibroblasts, but lactate-induced differentiation and the effects of GPR81 perturbation are more evident in nonfibrotic fibroblasts than in established IPF fibroblasts (Nho et al., 2023). These findings indicate that receptor abundance alone does not predict functional dependence on HCAR1/GPR81 signaling. Although HCAR1 has classically been viewed as a Gi/o-coupled lactate receptor, recent pharmacological profiling indicates a broader G-protein signaling repertoire, and whether such signaling properties operate similarly in IPF stromal or immune cells remains unresolved (Wel et al., 2022; Morland et al., 2015; Lind et al., 2026; Sakurai et al., 2014).

The pathological effects of extracellular lactate are therefore unlikely to reflect a single signaling route. MCT-mediated transport alters lactate flux, proton handling, and intracellular metabolic/redox states, whereas HCAR1/GPR81 perturbation interrogates receptor-mediated extracellular lactate sensing without necessarily altering lactate production or transport. These mechanisms should be experimentally and pharmacologically distinguished from lactate-associated extracellular acidification and intracellular lactylation. Moreover, because currently available HCAR1-directed pharmacological tools are less mature than MCT-directed interventions, genetic receptor perturbation and receptor-specific target-engagement measurements remain important for attributing phenotypes to HCAR1/GPR81. Target selection should therefore rely on functional perturbation and target-engagement evidence rather than transporter or receptor expression alone.

### 3.4 Lactate utilization and shuttle logic: mechanistic relevance and evidence boundaries

The transport relationships described above can be placed within the broader lactate-shuttle framework, in which lactate released from highly glycolytic cells can be taken up and utilized by neighboring or distant cells according to transmembrane lactate and proton gradients, cellular redox state, and metabolic demand. In this context, lactate is not solely a terminal glycolytic product or extracellular signaling metabolite, but can also function as a mobile carbon and redox intermediate. MCT1 is frequently associated with lactate uptake in relatively oxidative cells, whereas MCT4 often supports net export from highly glycolytic cells; however, neither transporter has an intrinsically fixed direction, and net flux remains gradient dependent (Ziehr et al., 2026; Broo and ks, 2018).

Following uptake, lactate can be reversibly converted to pyruvate by lactate dehydrogenase (LDH). In oxidative cellular states, LDH isoenzyme composition, substrate availability, and the NAD+/NADH ratio can favor lactate-to-pyruvate flux, with LDHB-enriched LDH activity often associated with lactate utilization. The resulting pyruvate can subsequently enter mitochondrial metabolism and contribute carbon to the tricarboxylic-acid (TCA) cycle. Stable-isotope studies in mammalian systems support lactate as a major oxidative carbon source, although such evidence provides general metabolic context rather than direct proof of intercellular lactate shuttling in IPF (Hui et al., 2017).

Pulmonary-fibrosis-relevant evidence supports the biological plausibility of this pathway. Ziehr et al. used 2 mM [U-^13^C_3_]-lactate and detected lactate-derived carbon in intracellular pyruvate and TCA-cycle intermediates in lung fibroblasts, demonstrating that extracellular lactate can contribute directly to cellular oxidative metabolism (Ziehr et al., 2026). Conversely, primary AEC2 cells isolated from human IPF lungs exhibit reduced oxidative metabolism, increased glycolytic lactate production, and a higher LDHA/LDHB ratio, while LDHA knockdown partially restores a more oxidative phenotype (Newt et al., 2021). These observations indicate that lactate production and utilization are strongly cell-state dependent in fibrotic lung disease.

Importantly, however, a complete fibroblast-to-macrophage lactate shuttle has not been demonstrated in human IPF. No study has yet shown isotope-resolved transfer of fibroblast-derived lactate into macrophages followed by direct quantification of its oxidation, TCA-cycle entry, incorporation into lactylated substrates, and subsequent macrophage-to-stromal feedback within the same disease-relevant system. Thus, lactate-shuttle biology provides an important metabolic framework for interpreting intercellular lactate exchange, but the strongest direct evidence for fibroblast–macrophage signaling in pulmonary fibrosis currently concerns lactate-responsive macrophage transcription and lactylation rather than demonstrated oxidative utilization of fibroblast-derived lactate by macrophages.

## 4 Lactate-mediated immunometabolic remodeling

### 4.1 Disease-associated macrophage states

Macrophage heterogeneity in fibrotic lung tissue reflects both cellular origin and microenvironmentally acquired functional states. Spatial and single-cell studies place disease-associated macrophage populations within or adjacent to fibrotic niches containing activated stromal and aberrant epithelial populations (Mayr et al., 2024; Joshi et al., 2020; Franzén et al., 2024; Morse et al., 2019). In human IPF, single-cell transcriptomic analysis has identified a proliferating macrophage population characterized by high SPP1 and MERTK expression and enrichment in fibrotic lung regions (Morse et al., 2019). Separately, lineage-tracing studies in experimental pulmonary fibrosis demonstrate that monocyte-derived alveolar macrophages can acquire profibrotic programs, localize near fibroblasts, and persist after recruitment to the injured lung (Joshi et al., 2020). These ontogenetic and transcriptional classifications are related but should not be treated as interchangeable. Accordingly, macrophage heterogeneity in IPF cannot be adequately represented by a binary M1/M2 framework, and this review uses the terms “disease-associated macrophage state” and “profibrotic functional state” when describing lactate-associated macrophage responses (Misharin et al., 2017; Philip et al., 2017; Bailey et al., 2024; King et al., 2024; Reyfman et al., 2019; Haberman et al., 2020).

Experimental pulmonary-fibrosis studies provide direct evidence that increased lactate availability can modify macrophage transcriptional outputs, although the quantitative and mechanistic resolution differs among experimental conditions. In Cui et al., human MRC5 fibroblasts were stimulated with 2 ng/mL TGF-β1 for 48 h, washed, and subsequently cultured in serum-free medium for an additional 48 h to generate myofibroblast-conditioned medium. This conditioned medium, as well as BALF obtained from fibrotic mice, increased expression of profibrotic mediators in bone-marrow-derived macrophages. To test lactate sufficiency more directly, BMDMs and primary murine alveolar macrophages were exposed to 20 mM lactate for 24 h, which increased expression of Arg1, Spp1/OPN, Pdgfa, Thbs1, and Vegfa and increased intracellular lactate and histone lactylation (Cui et al., 2021).

These experiments establish that macrophages are responsive to experimentally increased lactate and support a lactate-sensitive profibrotic transcriptional program. However, the 20 mM exposure should not be assumed to reproduce the concentration encountered by macrophages within human IPF lesions, because lesion-resolved human lactate concentrations have not been measured. In addition, conditioned medium and BALF are multicomponent biological fluids; their macrophage effects therefore cannot be attributed uniquely to lactate solely from these experiments. The study consequently provides mechanistic support for lactate responsiveness and fibroblast-to-macrophage signaling in experimental pulmonary fibrosis, but does not establish quantitatively defined fibroblast-derived lactate transfer within human IPF.

Macrophage persistence and survival during fibrosis are independently supported by lineage-tracing and survival-pathway studies. M-CSF/M-CSFR signaling sustains monocyte-derived alveolar macrophages within experimental fibrotic niches (Joshi et al., 2020), whereas recent work identifies TREM2 as a regulator of monocyte-derived alveolar macrophage survival and profibrotic activity, with TREM2 disruption promoting macrophage apoptosis and attenuating experimental fibrosis (Cui et al., 2025). However, current evidence does not establish that lactate directly regulates these persistence or survival pathways. A more evidence-consistent model is therefore that lactate reinforces selected profibrotic transcriptional outputs within macrophage states already shaped by ontogeny, cytokines, hypoxia, phagocytic activity, lipid metabolism, and the surrounding tissue microenvironment, rather than independently determining macrophage identity (King et al., 2024; Larson-Casey et al., 2019).

This distinction is important for pharmacological interpretation. SPP1, MERTK, ARG1, and related markers should not be considered lactate-specific readouts because their expression is influenced by macrophage origin and multiple microenvironmental signals. A reduction in a single conventional polarization marker after treatment should therefore not be interpreted as macrophage “repolarization.” Pharmacological evaluation of lactate-dependent macrophage reprogramming should instead combine multigene or state-level molecular readouts with lineage, spatial, and functional information, including downstream effects on fibroblast activation or matrix production. Where individual studies use M1/M2 terminology, these labels should be retained only when describing the original experimental polarization conditions and not treated as comprehensive macrophage states in IPF. Extracellular pH was not reported or comprehensively controlled across the exogenous-lactate and conditioned-medium experiments, so a contribution from extracellular acidification cannot be fully excluded from the observed macrophage responses.

### 4.2 Fibroblast-to-macrophage lactate signaling and candidate reciprocal interactions

The most direct lactate-specific evidence currently supports a stromal-to-immune signaling axis in which activated fibroblasts and myofibroblasts increase glycolysis and extracellular lactate release, thereby modifying macrophage transcriptional and epigenetic programs. In experimental pulmonary fibrosis, TGF-β1-induced myofibroblast-conditioned medium and exogenous lactate increase macrophage expression of profibrotic mediators including Arg1, Spp1, Pdgfa, Thbs1, and Vegfa, accompanied by increased histone lactylation (Cui et al., 2021). These experiments provide mechanistic support for a fibroblast-to-macrophage lactate axis, although the complete donor–recipient pathway has not yet been demonstrated using matched human IPF fibroblasts and macrophages.

Evidence for an immune-to-stromal metabolic arm is also available, although it has not been causally linked to fibroblast-derived lactate. In an LPS-driven pulmonary-fibrosis model, macrophage JNK-dependent TNF-α production increases PFKFB3-dependent glycolysis and lactate generation in lung fibroblasts, and interruption of this pathway attenuates experimental fibrosis (Xu et al., 2022). A recent non-lactate-specific study provides additional proof of principle that reciprocal immune–mesenchymal metabolic circuits can operate in lung fibrosis. Fibroblast P2RX4-dependent IL-6 signaling induces ARG1 activity in myeloid cells, generating ornithine that supports fibroblast proline and collagen synthesis (Yadav et al., 2025). Macrophage-specific Arg1 deletion attenuates this pathway in murine fibrosis, whereas ARG1 inhibition reduces collagen expression in human IPF precision-cut lung slices (Yadav et al., 2025). Importantly, ARG1-expressing myeloid cells were predominantly macrophages in mice but predominantly neutrophils in human IPF tissue, indicating that this pathway supports the biological feasibility of reciprocal metabolic crosstalk without establishing a human fibroblast–macrophage lactate feedback loop. Additional Tier B evidence from PM2.5-induced pulmonary fibrosis shows that macrophage-intrinsic lactate accumulation can promote H3K18 lactylation, suppress CHIP/Stub1 expression, stabilize TGF-β1, and enhance macrophage TGF-β1 secretion (Li J. et al., 2024; Liu et al., 2026). This provides a lactate-dependent mechanism through which macrophages may generate profibrotic outputs, but it remains unknown whether fibroblast-derived lactate initiates this pathway in IPF.

Taken together, these findings support a working model in which a mechanistically supported fibroblast-to-macrophage lactate axis could interface with independently established macrophage-to-stromal profibrotic pathways. Whether these directional interactions form a single closed lactate-dependent circuit in IPF remains unresolved. Spatial transcriptomic studies add anatomical plausibility by showing that disease-associated macrophages, activated stromal cells, and aberrant epithelial populations occupy neighboring fibrotic niches (Mayr et al., 2024; Franzén et al., 2024; Vannan et al., 2025). However, spatial proximity does not establish metabolite transfer, receptor engagement, or the direction of intercellular lactate flux. To date, oxygen tension, extracellular pH, lactate abundance, and donor–recipient lactate transfer have not been co-mapped at sufficient cellular resolution within the same human IPF lesions. Conditioned-medium experiments likewise cannot attribute recipient-cell effects uniquely to lactate without selective donor-cell perturbation and rescue.

Establishing a closed lactate-dependent circuit will require four complementary demonstrations within the same experimental framework: (i) donor-cell necessity, shown by fibroblast-specific inhibition of lactate production or export; (ii) lactate sufficiency or rescue, in which the recipient-cell phenotype is restored by controlled lactate replacement; (iii) recipient-cell specificity, demonstrated by macrophage-specific disruption of lactate uptake, sensing, or lactylation; and (iv) feedback closure, shown by a macrophage-dependent increase in fibroblast activation, matrix production, or tissue fibrosis. Ideally, these perturbations should be combined with isotope-resolved or spatial measurements demonstrating transfer of fibroblast-derived lactate to macrophages, followed by macrophage-specific molecular readouts and a functional stromal feedback endpoint. Without such perturbation-and-rescue evidence, concurrent increases in lactate, macrophage activation, and fibrosis should be interpreted as support for linked processes rather than proof of a complete bidirectional lactate circuit.

### 4.3 Histone lactylation and transcriptional reprogramming

Histone lactylation provides a mechanism through which altered lactate metabolism may be coupled to transcriptional regulation in recipient cells. The original description of histone lactylation established that glycolysis-derived lactate can increase lysine L-lactylation on histones and influence chromatin-associated transcriptional activity (Zhang et al., 2019). In the Cui et al. experimental system, 20 mM lactate for 24 h increased intracellular lactate and histone lysine lactylation in BMDMs, while myofibroblast-conditioned medium produced a similar increase in intracellular lactate and histone lactylation (Cui et al., 2021). Chromatin immunoprecipitation after lactate exposure demonstrated increased lactyl-lysine enrichment at the proximal promoter regions of Arg1, Pdgfa, Thbs1, and Vegfa, in parallel with increased transcription of these genes (Cui et al., 2021). These findings provide locus-associated evidence linking lactate exposure to profibrotic macrophage transcription, although the specific lactylated histone residues responsible for these effects were not resolved. p300 knockdown reduces lactate- and myofibroblast-conditioned-medium-induced histone lactylation, promoter-associated lactylation, and profibrotic gene expression, supporting a contribution of p300 to this pathway (Cui et al., 2021). The ChIP strategy used a pan–lactyl-lysine antibody and therefore provides locus-associated evidence for lactylation but does not identify a specific histone residue or establish that the detected promoter-associated signal is sufficient, by itself, to drive the complete macrophage phenotype (Cui et al., 2021). However, because p300 is also a major histone acetyltransferase and transcriptional coactivator, p300 perturbation does not selectively isolate lactylation from its broader chromatin-regulatory functions.

In PM2.5-induced pulmonary fibrosis, increased macrophage lactate production is associated with enrichment of H3K18 lactylation (H3K18la) at the Stub1 promoter, reduced CHIP/Stub1 expression, impaired TGF-β1 degradation, and increased macrophage TGF-β1 secretion. Inhibition of lactate production attenuates these molecular changes and reduces experimental fibrosis (Liu et al., 2026). More recent Tier B studies provide residue- and locus-specific evidence for histone lactylation, although each derives from an etiologically distinct experimental fibrosis model. In Nd_2_O_3_-induced pulmonary fibrosis, macrophage H4K12 lactylation was linked to PIK3R2/PI3K/Akt/mTOR signaling and profibrotic macrophage–fibroblast communication (Li W. et al., 2026). However, the use of 2-DG broadly perturbs glycolysis and therefore does not selectively isolate lactate or H4K12 lactylation as the causal mediator. In silica-nanoparticle-induced fibrosis, H3K18la was linked to NOS2-associated macrophage inflammatory programming and fibroblast activation (Shi et al., 2025); nevertheless, the toxicant model and conventional M1-polarization framework limit direct extrapolation to disease-associated macrophage states in human IPF.

The enzymology of lysine lactylation has also expanded beyond the initial p300-centered framework. AARS1 and AARS2 were recently identified as intracellular L-lactate sensors and ATP-dependent lysine lactyltransferases capable of modifying broad protein substrates (Li H. et al., 2024). These enzymes activate L-lactate through a lactate-AMP intermediate before transfer of the lactyl group to lysine residues, with AARS2-mediated cGAS lactylation providing a functionally validated biochemical example (Li H. et al., 2024). This discovery broadens the biochemical routes through which intracellular lactate can regulate protein function, but it should not yet be extrapolated to the histone-lactylation machinery operating in pulmonary macrophages. Direct roles for AARS1 or AARS2 in macrophage histone lactylation, fibroblast–macrophage crosstalk, or human IPF have not been demonstrated; their relevance therefore remains Tier C mechanistic evidence rather than an established component of the IPF lactylation pathway.

Lactylation should be experimentally distinguished from MCT-mediated lactate transport, HCAR1/GPR81-mediated extracellular sensing, and lactate-associated acidification because these mechanisms may operate concurrently but are not mechanistically interchangeable. An increase in pan-histone or total H3 lysine lactylation indicates altered bulk lactylation but does not identify the responsible histone residue, genomic locus, lactylation isomer, or causal target gene. Analytical specificity is also important because lysine L-lactylation is chemically distinct from D-lactylation and N-ε-(carboxyethyl)-lysine; these species should therefore be assigned only with validated analytical methods. Stronger causal attribution therefore requires integration of residue-specific lactylation measurements, locus-resolved chromatin analysis, targeted proteomics where feasible, target-gene perturbation, and functional rescue.

These considerations are equally important for pharmacological interpretation. Interventions targeting p300 or upstream lactate metabolism can alter multiple metabolic and chromatin processes in addition to histone lactylation, and apparent normalization of H3Kla alone should not be treated as proof of target-specific antifibrotic action. Pharmacological studies should demonstrate target engagement, residue- or locus-relevant lactylation changes, downstream transcriptional consequences, and functional antifibrotic benefit while assessing effects on basal macrophage viability and immune function. At present, histone lactylation is therefore better positioned as an emerging mechanistic axis and candidate pharmacodynamic readout than as a pharmacologically validated stand-alone therapeutic target in IPF.

### 4.4 Spatially organized profibrotic niches

Human spatial studies identify anatomically restricted but heterogeneous fibrotic neighborhoods rather than a single uniform profibrotic niche. In IPF, myofibroblast- and aberrant-basaloid-cell-enriched fibrotic regions can occur adjacent to airway macrophage niches containing SPP1-expressing macrophages (Mayr et al., 2024). Other spatial analyses identify activated fibrotic fibroblast states expressing CTHRC1 and FAP within remodeled regions and progressive accumulation of SPP1-expressing macrophages with increasing pathological remodeling (Morse et al., 2019; Vannan et al., 2025). Comparative spatial transcriptomic studies further demonstrate substantial heterogeneity among epithelial, stromal, and immune compartments in human IPF and experimental pulmonary fibrosis (Franzén et al., 2024). These spatial associations establish anatomical opportunities for intercellular communication but do not demonstrate direct metabolic coupling between these cell states (Reyfman et al., 2019; Haberman et al., 2020).

When integrated with separate mechanistic studies showing fibroblast lactate production and macrophage lactate-responsive reprogramming, these spatial observations support a testable model of localized lactate-associated communication (Yan et al., 2024a; Cui et al., 2021). However, this interpretation remains inferential because the spatial and metabolic components have not been demonstrated simultaneously within the same human IPF lesions. Current human spatial studies do not simultaneously resolve lactate abundance, extracellular pH, oxygen tension, and cell-specific lactate production or sensing. Spatial proximity therefore cannot establish the cellular source of lactate, the direction of lactate flux, receptor or transporter engagement in recipient cells, or lactate-dependent functional causality. Bulk tissue, BALF, and circulating lactate measurements average across heterogeneous compartments and may therefore fail to capture short-range metabolic gradients within active fibrotic regions. Resolving these gradients will require integration of spatial transcriptomics or multiplexed cellular imaging with direct metabolite measurements, such as spatial metabolomics or imaging mass spectrometry, together with spatial assessment of hypoxia, extracellular acidification, and cell-specific lactate transport or sensing. Where feasible, isotope-resolved tracing in tissue-preserving *ex vivo* systems could further distinguish lactate production from intercellular transfer and recipient-cell utilization. For lactate-directed therapies, pharmacodynamic assessment should therefore prioritize target engagement within fibrotic lesions and relevant cellular neighborhoods rather than relying solely on circulating or whole-lung lactate concentrations. Such approaches will be necessary to determine whether lactate-associated niches represent reproducible and therapeutically distinct compartments in IPF.

These observations form the basis for the proposed spatial model shown in Figure 2, in which lactate, oxygen, and pH gradients are explicitly presented as conceptual rather than directly demonstrated features of human IPF lesions.

**FIGURE 2:**
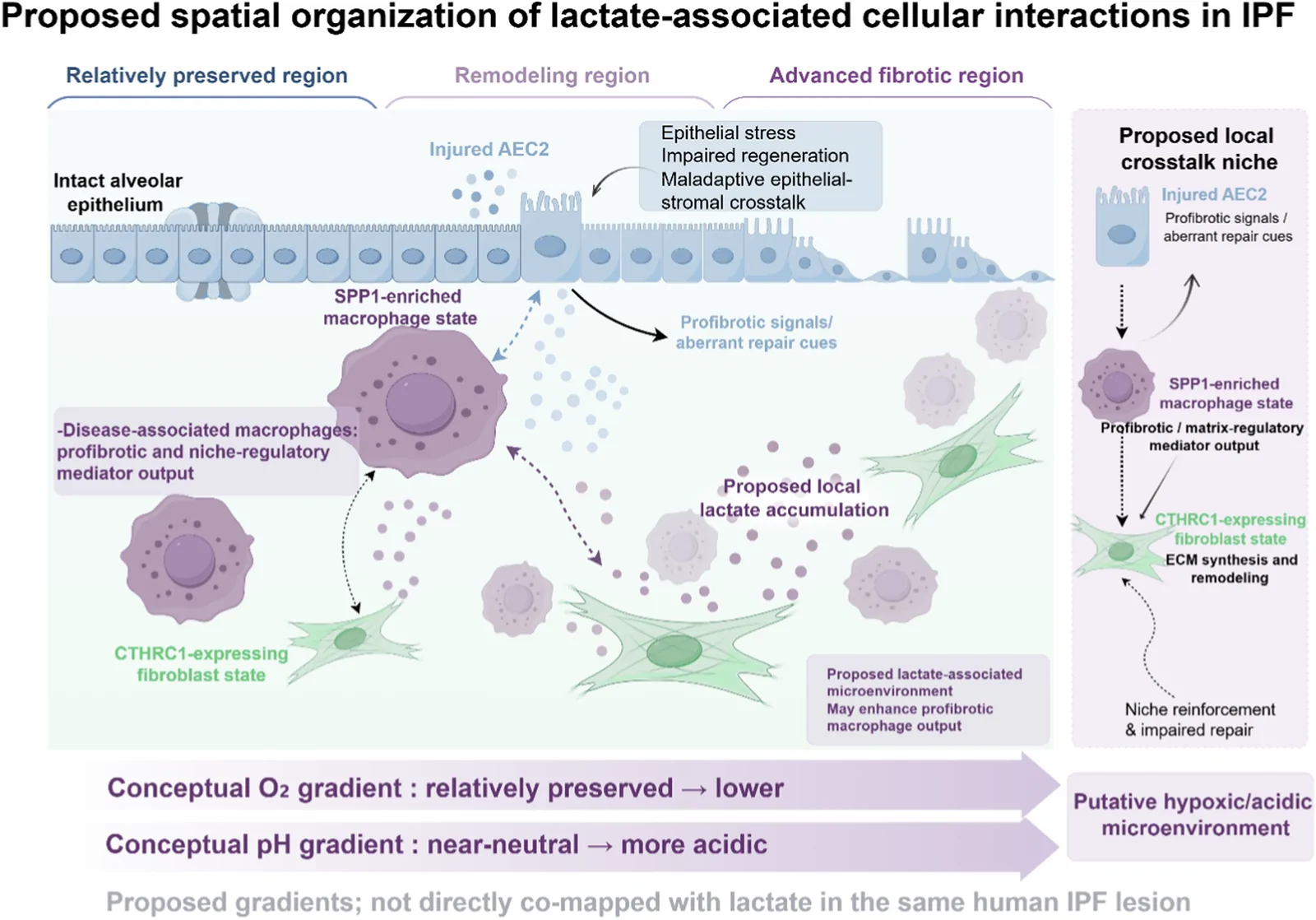
Proposed spatial organization of lactate-associated cellular interactions in idiopathic pulmonary fibrosis. Human spatial studies identify heterogeneous fibrotic neighborhoods containing injured or aberrant epithelial populations, activated fibroblast states, and disease-associated macrophage populations. SPP1-enriched macrophage states and CTHRC1-expressing fibroblast states are associated with remodeled fibrotic regions, providing anatomical opportunities for intercellular communication. Integration of these spatial observations with separate mechanistic evidence of fibroblast glycolysis, lactate release, macrophage lactate-responsive transcription, and HIF-associated signaling supports a testable model of localized lactate-associated cellular interactions. However, lactate abundance, extracellular pH, oxygen tension, and donor–recipient lactate flux have not been directly co-mapped within the same human IPF lesions. Spatial proximity should therefore be interpreted as biological plausibility rather than evidence of direct lactate transfer or lactate-dependent causality. Solid arrows denote comparatively well-supported spatial or biological relationships; dashed arrows indicate proposed or incompletely validated lactate-associated interactions. The depicted oxygen, pH, and lactate gradients are conceptual and non-quantitative rather than directly measured spatial gradients in human IPF lesions.

### 4.5 Non-macrophage immune cells: disease relevance and limits of lactate-specific evidence

Non-macrophage immune cells also occupy fibrotic lung niches and may respond to altered lactate availability, but their disease relevance should be distinguished from direct lactate-specific causality. Human IPF studies identify altered T-cell composition and functional states, including disease-associated CD8^+^ T-cell populations with distinct transcriptional and metabolic features (Wei et al., 2024). In non-IPF settings, lactate and extracellular acidification can suppress effector T-cell proliferation, cytokine production, and cytotoxic function while supporting selected regulatory programs. However, equivalent lactate-dependent mechanisms have not been demonstrated directly in IPF T cells. These observations should therefore be regarded as Tier C mechanistic context rather than evidence of an established lactate–T-cell pathway in IPF (Brand et al., 2016).

Natural killer (NK) cells provide a similar example of disease relevance without established lactate causality. Human IPF lungs show reduced NK-cell abundance or activity, and the end-stage IPF microenvironment can impair NK-cell function (Cruz et al., 2021; Cruz et al., 2023). Lactate can suppress NK-cell metabolism, cytotoxicity, and cytokine production in other disease contexts (Brand et al., 2016), but current IPF studies do not identify lactate as the responsible mediator. Accordingly, NK-cell dysfunction should not be incorporated into an IPF lactate-signaling model without direct metabolic perturbation or rescue.

Neutrophils are also relevant to fibrotic lung disease. In human IPF, peripheral neutrophil levels correlate with the extent and subsequent progression of radiographic fibrosis (Achaiah et al., 2023), and recent work on ARG1-dependent profibrotic metabolism indicates that ARG1-expressing myeloid cells in human IPF tissue are predominantly neutrophils rather than macrophages (Yadav et al., 2025). However, the ARG1 pathway is ornithine/proline dependent rather than lactate dependent.

Group 2 innate lymphoid cells (ILC2s) represent another immune population with experimental relevance to pulmonary fibrosis. IL-33/ST2-dependent recruitment and activation of ILC2s promotes IL-13-associated fibroblast activation and fibrosis in bleomycin models (Zhao et al., 2018). Conversely, tumor-derived lactic acid can suppress ILC2 survival and function in a non-pulmonary disease context, illustrating that lactate effects on ILC2s are highly context dependent. No comparable lactate–ILC2 mechanism has yet been demonstrated in pulmonary fibrosis or human IPF (Wagner et al., 2020).

Taken together, non-macrophage immune populations are clearly relevant to fibrotic lung biology, but lactate-specific causal evidence remains substantially weaker than for pulmonary macrophages. Macrophages therefore remain the immune compartment with the most direct pulmonary-fibrosis evidence linking increased lactate availability to defined transcriptional and lactylation-associated outputs.

## 5 Downstream fibrotic consequences

### 5.1 Lactate-associated acidification and TGF-β reinforcement

Kottmann et al. provided the most direct quantitative evidence linking lactic acid-associated acidification to profibrotic TGF-β activation. In whole-lung homogenates from patients with IPF and non-fibrotic controls (n = 6 per group), lactic acid was quantified using ^1H-PASS NMR and was significantly increased in IPF lung tissue (Kottmann et al., 2012). In primary human lung fibroblast experiments, exogenous lactic acid was tested at 1, 10, and 20 mM. Addition of these concentrations rapidly reduced culture-medium pH to approximately 7.2, 6.7, and 6.2, respectively, from a baseline near 7.8. One millimolar lactic acid produced little myofibroblast differentiation, 10 mM produced a response comparable to TGF-β stimulation, and 20 mM produced a stronger differentiation response, accompanied by increased α-SMA, calponin, and collagen-associated readouts (Kottmann et al., 2012).

Importantly, the study incorporated pH-manipulation experiments in which lactic-acid-containing medium was adjusted toward neutral/alkaline culture conditions, supporting the interpretation that activation of latent TGF-β and myofibroblast differentiation depended substantially on extracellular acidification. Accordingly, this study provides strong evidence for a lactic-acid/acidification–TGF-β mechanism, but it should not be interpreted as demonstrating that the lactate anion itself, HCAR1/GPR81 signaling, or lactylation mediated the observed fibroblast phenotype.

Lactic acid, lactate salts, and extracellular acidification should therefore not be treated as mechanistically interchangeable experimental conditions. A phenotype observed after lactic acid exposure may reflect the lactate anion, proton-associated extracellular acidification, altered MCT-mediated lactate/proton transport, or a combination of these processes. Mechanistic studies should independently control extracellular lactate and pH and, where TGF-β activation is implicated, distinguish active from total TGF-β. Parallel perturbation of HCAR1/GPR81 and MCT-dependent transport is also required before receptor-mediated sensing, transmembrane exchange, or acidification can be assigned as the dominant mechanism. These distinctions are pharmacologically important because reducing lactate production, blocking lactate transport, and modifying extracellular acidity are not equivalent interventions.

### 5.2 Fibroblast activation and matrix remodeling

Activated fibroblasts and myofibroblasts represent a major downstream effector compartment for lactate-associated signaling in pulmonary fibrosis. Increased glycolytic flux and lactate accumulation accompany profibrotic fibroblast activation, while experimental manipulation of lactate production and transport alters myofibroblast differentiation and matrix-associated phenotypes. Recent genetic and pharmacological studies of MCT1 and MCT4 provide particularly strong evidence that lactate transport is functionally required for TGF-β1-induced myofibroblast differentiation. MCT inhibition shifts fibroblast metabolism toward oxidative phosphorylation and attenuates experimental pulmonary fibrosis, with MCT4-directed inhibition showing antifibrotic activity in both young and aged mice (Ziehr et al., 2026). Experimental Akt2–PDK1 signaling has been directly linked to altered pyruvate partitioning, lactate accumulation, and pulmonary fibrosis (Sun et al., 2024a). Complementary studies indicate that fibroblast glycolytic and lactate-producing metabolism can also be regulated through PI3K–Akt–mTOR/PFKFB3 signaling (Hu et al., 2020) and the alamandine/MrgD axis (Wang et al., 2023), highlighting multiple upstream routes by which profibrotic fibroblast metabolism can be modified.

Single-cell and spatial studies identify CTHRC1-expressing fibroblasts as prominent matrix-producing states in fibrotic human lung tissue. However, no current evidence demonstrates that lactate directly induces or maintains the CTHRC1-expressing state. Lactate-associated metabolism should therefore be viewed as one component of a broader network involving TGF-β signaling, matrix-derived mechanical cues, and fibroblast-state regulation rather than as a defining determinant of this population.

Importantly, reduced α-SMA expression alone should not be interpreted as reversal of a fibrotic fibroblast state. More convincing evidence would demonstrate sustained reduction of collagen synthesis and matrix remodeling, restoration of apoptosis susceptibility or metabolic homeostasis, and persistence of these effects after withdrawal of the profibrotic stimulus. *In vivo*, therapeutic relevance is strengthened when treatment is initiated after fibrosis is established and improves matrix burden, tissue mechanics, or pulmonary function rather than only preventing early injury. Current evidence therefore supports lactate-related pathways as regulators of fibroblast activation and matrix remodeling, whereas a specific requirement for lactate in long-term fibroblast persistence remains unresolved.

### 5.3 Alveolar epithelial injury and maladaptive repair

Human IPF-derived primary AEC2 cells exhibit impaired oxidative metabolism, increased reliance on glycolytic lactate production, and altered LDHA/LDHB balance, establishing disease-relevant epithelial metabolic dysregulation (Newt et al., 2021). However, these observations do not determine whether lactate dysregulation precedes epithelial injury or develops secondarily to cellular stress. Experimental evidence suggests that elevated lactate can contribute to epithelial stress and apoptosis. In bleomycin-induced pulmonary fibrosis and complementary alveolar epithelial-like cell experiments, exposure to 10 mM lactate for 48 h activated an ATF4–CHOP-associated endoplasmic-reticulum stress response and increased apoptotic signaling (Sun et al., 2024b). Because the mechanistic cellular experiments were not performed in primary human IPF AEC2 cells, this pathway should be considered experimental rather than a validated human IPF epithelial mechanism.

Additional Tier B evidence from an LPS-driven fibrosis model indicates that disrupted epithelial MCT1-dependent lactate handling is associated with lactate accumulation and EMT-like epithelial plasticity (Feng et al., 2024). In complementary epithelial-cell experiments, exogenous lactate was tested at 10 and 20 mM for up to 48 h, with the higher exposure producing more pronounced EMT-like phenotypic changes (Feng et al., 2024). Because these findings derive from an inflammation-dominant experimental model rather than human IPF, they should be interpreted as evidence that disturbed lactate transport can modify epithelial identity, not as proof of complete epithelial-to-mesenchymal lineage conversion in IPF.

Independent human and *ex vivo* studies demonstrate that fibroblast-derived signals, including TGF-β1/sFRP2-associated noncanonical Wnt signaling, can alter epithelial cell state and promote metaplastic remodeling in IPF (Cohen et al., 2024). These findings establish the broader biological importance of fibroblast-to-epithelial communication but do not implicate lactate as the initiating mediator. When considered together with experimental evidence of lactate-associated epithelial stress and acidification-dependent TGF-β activation, they support a hypothesis that disturbed lactate homeostasis could amplify maladaptive epithelial–stromal communication rather than initiate it.

A major unresolved question is therefore whether epithelial lactate dysregulation precedes epithelial injury, amplifies an already established stress response, or develops as a downstream consequence of epithelial dysfunction. Acute exposure to 10–20 mM exogenous lactate in epithelial-cell models may not reproduce the chronic, spatially heterogeneous lactate and pH conditions encountered within human IPF lesions. Resolving this question will require primary human AEC2 or patient-relevant alveolar organoid systems with cell-specific manipulation of lactate production, transport, or sensing, coupled to regenerative endpoints such as survival, differentiation, barrier restoration, and epithelial–stromal communication. Such studies would help distinguish lactate as an initiating mediator, a disease amplifier, or a downstream metabolic consequence.

## 6 Immunopharmacological opportunities

The therapeutic relevance of lactate biology depends not only on mechanistic involvement but also on target selectivity, demonstrable target engagement, efficacy in established fibrosis, and preservation of physiological lactate metabolism. Importantly, mechanistic importance should not be equated with pharmacological tractability, because a biologically central node may still represent a poor therapeutic target if selective modulation or an acceptable safety window cannot be achieved. No lactate-directed intervention has yet demonstrated clinical therapeutic efficacy in IPF. Accordingly, the targets discussed below are prioritized according to disease relevance and pharmacological maturity rather than mechanistic plausibility alone.

### 6.1 Targeting lactate production

Among upstream regulators of lactate-producing metabolism, PFKFB3 currently has comparatively strong pathway-level evidence in pulmonary fibrosis. Anlotinib suppresses PCBP3-dependent PFKFB3 translation, reduces TGF-β1-induced glycolysis and lactate accumulation in myofibroblasts, and attenuates bleomycin-induced fibrosis (Chen et al., 2021). Albendazole has similarly been reported to suppress myofibroblast aerobic glycolysis through an LRRN3/PFKFB3-associated mechanism and to reduce fibrotic responses in experimental models and human precision-cut lung slices (Zeng et al., 2024). However, neither intervention constitutes selective PFKFB3 pharmacology: anlotinib is a multitarget tyrosine-kinase inhibitor, whereas albendazole has broad repurposed pharmacology. These studies therefore establish PFKFB3 as a mechanistically supported pathway node but do not demonstrate that selective PFKFB3 inhibition is sufficient to reproduce the observed antifibrotic effects.

LDHA occupies a more proximal position in lactate generation by catalyzing pyruvate-to-lactate conversion. Increased LDH5, the LDHA-predominant tetrameric isoenzyme, in human IPF lung tissue and an increased LDHA/LDHB ratio in IPF-derived AEC2 cells provide direct disease-relevant evidence of altered LDH-dependent metabolism (Newt et al., 2021; Kottmann et al., 2012). Genetic manipulation of LDHA can modify lactate production in disease-relevant cellular systems, but selective LDHA-directed pharmacology has not yet been adequately validated in established pulmonary fibrosis. LDHA therefore has strong mechanistic relevance but remains pharmacologically immature.

Pyruvate dehydrogenase kinase 1 (PDK1; not PDPK1) regulates pyruvate partitioning by inhibiting the pyruvate dehydrogenase complex and thereby limiting mitochondrial pyruvate oxidation. Experimental Akt2–PDK1 signaling has been linked to lactate accumulation and pulmonary fibrosis (Sun et al., 2024a), supporting a functional role for altered pyruvate handling. However, pulmonary-fibrosis-specific evidence for selective PDK1 inhibition remains limited, and PDK isoforms contribute broadly to physiological fuel selection and metabolic adaptation. PDK1 should therefore be regarded as a mechanistically relevant but pharmacologically immature node rather than a validated antifibrotic target.

Because glycolysis and lactate production support normal cellular metabolism, production-directed strategies face an intrinsic selectivity challenge. Advancement should require target-specific perturbation, direct demonstration of reduced pathological lactate flux, efficacy when treatment is initiated after fibrosis establishment, and preservation of systemic metabolic function. On current evidence, PFKFB3 has comparatively strong pathway-level proof of concept among production-associated nodes, although selective PFKFB3-dependent antifibrotic efficacy remains insufficiently established. LDHA and PDK1 likewise remain insufficiently validated with selective antifibrotic pharmacology.

### 6.2 Targeting transport and sensing

Among the lactate-associated targets considered here, fibroblast-associated MCT4 currently has the most developed preclinical evidence package. Genetic and pharmacological inhibition of MCT4 reduces TGF-β1-induced myofibroblast differentiation and attenuates experimental pulmonary fibrosis, while the MCT4-directed inhibitor VB253 has demonstrated antifibrotic activity in both young and aged mice (Ziehr et al., 2026). Complementary studies in patient-derived IPF fibroblasts show that hypoxia increases MCT4 expression and extracellular lactate release and that MCT4 silencing or pharmacological inhibition reduces extracellular lactate accumulation and profibrotic paracrine signaling (Nho et al., 2026). Together, these findings provide convergent evidence of disease relevance, target perturbation, target engagement, and *in vivo* efficacy. This positioning should nevertheless be considered provisional because the supporting pharmacological evidence remains preclinical and requires independent validation. Nevertheless, independent confirmation of compound selectivity, pharmacokinetics, systemic safety, and efficacy alongside contemporary antifibrotic therapy is required before MCT4 can be considered clinically validated (Wel et al., 2022; Morland et al., 2015; Zhang et al., 2024; Sakurai et al., 2014).

MCT1 presents a substantially more context-dependent pharmacological problem. Because MCT1 can support lactate uptake or export according to cell type and metabolic gradients, generalized inhibition may disrupt physiological lactate utilization as well as pathological exchange. Consistent with this concern, experimental reduction of epithelial MCT1 in an LPS-driven fibrosis model was associated with lactate accumulation and EMT-like epithelial changes (Feng et al., 2024). Although this finding should not be extrapolated directly to human IPF, it illustrates why MCT1-directed therapy requires cell-type-specific target engagement and flux measurements rather than reliance on transporter expression alone.

Human oncology experience with AZD3965 nevertheless provides important information on the translational tractability of MCT1 inhibition. In a first-in-human phase I study, oral AZD3965 achieved target-engaging systemic exposures, and 10 mg twice daily was selected as the recommended phase II dose. Dose-limiting toxicities at higher exposures included predominantly reversible ocular toxicity together with isolated troponin elevation and acidosis. These findings demonstrate that systemic MCT1 inhibition is pharmacologically achievable in humans, but they also underscore the physiological liabilities of generalized MCT1 blockade. Importantly, AZD3965 has not demonstrated antifibrotic efficacy and has not been clinically evaluated in IPF; the oncology data therefore inform pharmacological tractability rather than disease-specific therapeutic validation (Halford et al., 2023).

HCAR1/GPR81 represents a mechanistically distinct but less pharmacologically mature strategy. L-lactate is the endogenous HCAR1/GPR81 ligand, and pharmacological studies have identified receptor-active tools including 3,5-dihydroxybenzoic acid and more recently characterized agonist or allosteric compounds such as AZ7136, AZ2114, and GPR81 agonist 1 (Lind et al., 2026; Sakurai et al., 2014). In IPF-derived mesenchymal progenitor cells, GPR81 knockdown provides direct disease-relevant evidence that receptor-dependent lactate sensing contributes to profibrotic behavior (Yang et al., 2023), while fibroblast paracrine studies provide complementary evidence of donor-MCT4/recipient-HCAR1 signaling (Nho et al., 2023; Nho et al., 2026).

In contrast to the expanding agonist pharmacology, a rigorously validated selective HCAR1/GPR81 antagonist remains unavailable. Compounds such as 3-hydroxybutyrate/3-OBA that have previously been used as presumed antagonists should therefore not be treated as receptor-selective blockers (Mohammad et al., 2022). Genetic receptor perturbation currently provides stronger causal evidence than antagonist-based pharmacology in IPF-relevant systems. HCAR1/GPR81 should consequently remain a mechanistically compelling target for tool development rather than a near-term therapeutic candidate.

Taken together, current evidence provisionally favors MCT4-targeted lactate-transport modulation, particularly in fibroblast-associated disease contexts, over generalized MCT1 inhibition or HCAR1/GPR81-directed pharmacology.

### 6.3 Targeting macrophage reprogramming and lactylation

Lactate-responsive macrophage transcription provides a downstream intervention point within the experimentally supported fibroblast-to-macrophage lactate axis. Myofibroblast-derived lactate increases histone lactylation and profibrotic transcriptional outputs in pulmonary macrophages (Cui et al., 2021), suggesting that intervention could occur upstream by reducing pathological lactate delivery or downstream by modifying lactate-responsive chromatin regulation (Zhang et al., 2024; Liu et al., 2026; Li W. et al., 2026; Shi et al., 2025).

In pulmonary macrophages, p300 knockdown reduces lactate-induced histone lactylation and profibrotic transcription (Cui et al., 2021), supporting a functional contribution of p300-dependent chromatin regulation. However, p300 is a broad acetyltransferase and transcriptional coactivator, and its inhibition cannot selectively isolate lactylation from other chromatin programs. p300 should therefore currently be regarded primarily as a mechanistic probe of lactylation-associated chromatin regulation rather than as a lactylation-selective therapeutic target. Recent residue- and locus-specific studies in non-IPF pulmonary-fibrosis models (Liu et al., 2026; Li W. et al., 2026; Shi et al., 2025) strengthen the biological plausibility of lactylation-directed intervention. However, these studies use etiologically distinct Nd_2_O_3_-, silica-nanoparticle-, or PM2.5-driven fibrosis models, and several upstream interventions broadly perturb glycolysis rather than selectively inhibiting the implicated lactylation event. Complementary macrophage evidence from non-pulmonary disease contexts, including MCT4-dependent H3K18 lactylation in atherosclerosis (Zhang et al., 2024), provides additional mechanistic support but should be regarded as Tier C evidence for IPF.

AARS1 and AARS2 have expanded the enzymological landscape by providing ATP-dependent lysine lactyltransferase activity and intracellular lactate sensing. However, neither enzyme has been causally implicated in pulmonary macrophage histone lactylation or human IPF. They should therefore remain Tier C target-discovery candidates rather than entries in the current pulmonary-fibrosis therapeutic pipeline.

At present, lactylation is more compelling as a mechanistic and pharmacodynamic readout than as a stand-alone therapeutic target. Pharmacodynamic assessment should combine cell-resolved lactylation with macrophage transcriptional outputs and functional stromal or tissue endpoints, because reductions in whole-lung H3Kla may reflect changes in cellular composition rather than target-specific modulation within macrophages. Therapeutic strategies should therefore aim to restrict pathological lactate-responsive macrophage outputs rather than broadly deplete or suppress macrophages, whose physiological functions in host defense, injury clearance, and tissue repair must be preserved.

### 6.4 Direct extracellular lactate elimination

Direct removal of extracellular lactate represents a mechanistically distinct intervention from suppressing lactate production, blocking MCT-mediated transport, inhibiting HCAR1/GPR81 signaling, or targeting downstream lactylation. A recent proof-of-concept study developed a lactate-scavenging nanoplatform, CeO_2_@PLP, consisting of phosphatidylserine–polyethylene-glycol-modified hollow mesoporous CeO_2_ nanoparticles co-loaded with lactate oxidase (LOx) and pirfenidone. The platform was designed to accumulate in fibrotic lung tissue through TREM2-associated targeting and to couple LOx-mediated lactate degradation with CeO_2_-dependent reactive-oxygen-species scavenging and pirfenidone-mediated antifibrotic activity. In bleomycin-induced pulmonary fibrosis, CeO_2_@PLP reduced lactate accumulation, attenuated macrophage profibrotic polarization-associated responses and fibroblast differentiation, and improved fibrotic outcomes (Xu et al., 2025).

This study is important because it establishes extracellular lactate depletion itself as an experimentally tractable therapeutic concept rather than treating lactate only through upstream metabolic inhibition. However, the evidence should be considered Tier B and interpreted cautiously. CeO_2_@PLP is a multicomponent intervention in which LOx-mediated lactate consumption, CeO_2_-mediated ROS control, TREM2-associated delivery, and pirfenidone each have the potential to influence fibrosis independently. Consequently, the total antifibrotic effect cannot be attributed uniquely to extracellular lactate elimination. In addition, efficacy has so far been demonstrated in experimental fibrosis rather than human IPF, and the safety, pharmacokinetics, lesion-level target engagement, and long-term consequences of enzymatically manipulating extracellular lactate remain unresolved. Direct lactate elimination should therefore be viewed as an emerging proof-of-concept strategy rather than a clinically validated therapeutic approach.

### 6.5 Combination strategies, biomarkers, and translational models

Lactate-directed therapies should be developed as potential additions to the contemporary IPF therapeutic landscape rather than as replacements for approved treatment. Pirfenidone and nintedanib remain established antifibrotic agents, while the preferential PDE4B inhibitor nerandomilast has expanded approved pharmacological options following phase III validation and regulatory approval. FIBRONEER-IPF included patients with and without background nintedanib or pirfenidone therapy, reinforcing the relevance of evaluating new mechanistically distinct interventions against contemporary background treatment rather than in isolation (Maher et al., 2025). For lactate-directed combinations, mechanistic complementarity should not be equated with clinical benefit; development should demonstrate incremental antifibrotic efficacy, pharmacodynamic target engagement, and acceptable cumulative toxicity when added to approved therapy.

Pharmacodynamic biomarkers should be organized across three complementary levels. First, target-engagement measures should confirm modulation of the intended lactate-production, transport, sensing, or lactylation pathway in disease-relevant cells or tissue. Second, cell-state measures should demonstrate corresponding changes in fibroblast, macrophage, or epithelial functional programs. Third, tissue-level endpoints should establish reduced matrix remodeling or improved physiological function. A reduction in bulk lactate concentration, transporter expression, or H3Kla alone should not be considered evidence of antifibrotic efficacy.

A rational translational sequence would progress from cell-specific perturbation in primary IPF cells, through donor–recipient co-culture systems, to organoids or precision-cut lung slices that preserve multicellular interactions, followed by established-fibrosis *in vivo* models incorporating pharmacokinetic, target-engagement, and safety assessment. Human-derived systems are most informative when they confirm target dependence and functional antifibrotic activity rather than merely reproduce expression changes observed in simplified models.

Patient stratification is unlikely to be achieved using circulating lactate alone because systemic lactate is strongly influenced by exercise, hepatic and renal handling, and whole-body metabolism. Candidate enrichment strategies may instead require multimodal signatures incorporating lesion-associated glycolytic activity, MCT4 enrichment, hypoxia-related features, and relevant stromal or immune states (Yan et al., 2024a; Nho et al., 2023; Nho et al., 2026; Mayr et al., 2024). These features remain exploratory rather than validated clinical biomarkers. A clinically useful strategy will therefore likely require integration of accessible biomarkers with imaging, tissue-informed signatures, and longitudinal functional outcomes.

Overall, translational advancement should be based on convergence of target engagement, functional antifibrotic efficacy, therapeutic-stage activity, and an acceptable safety window rather than pathway prominence alone. This framework currently favors MCT4-targeted modulation of lactate transport, particularly in fibroblast-associated disease contexts, while placing production-, receptor-, and lactylation-directed strategies at earlier stages of pharmacological validation.

The resulting qualitative translational positioning of lactate-associated targets is summarized in Figure 3. The matrix reflects current human disease relevance and pharmacological tractability rather than a numerical score or clinical recommendation.

**FIGURE 3:**
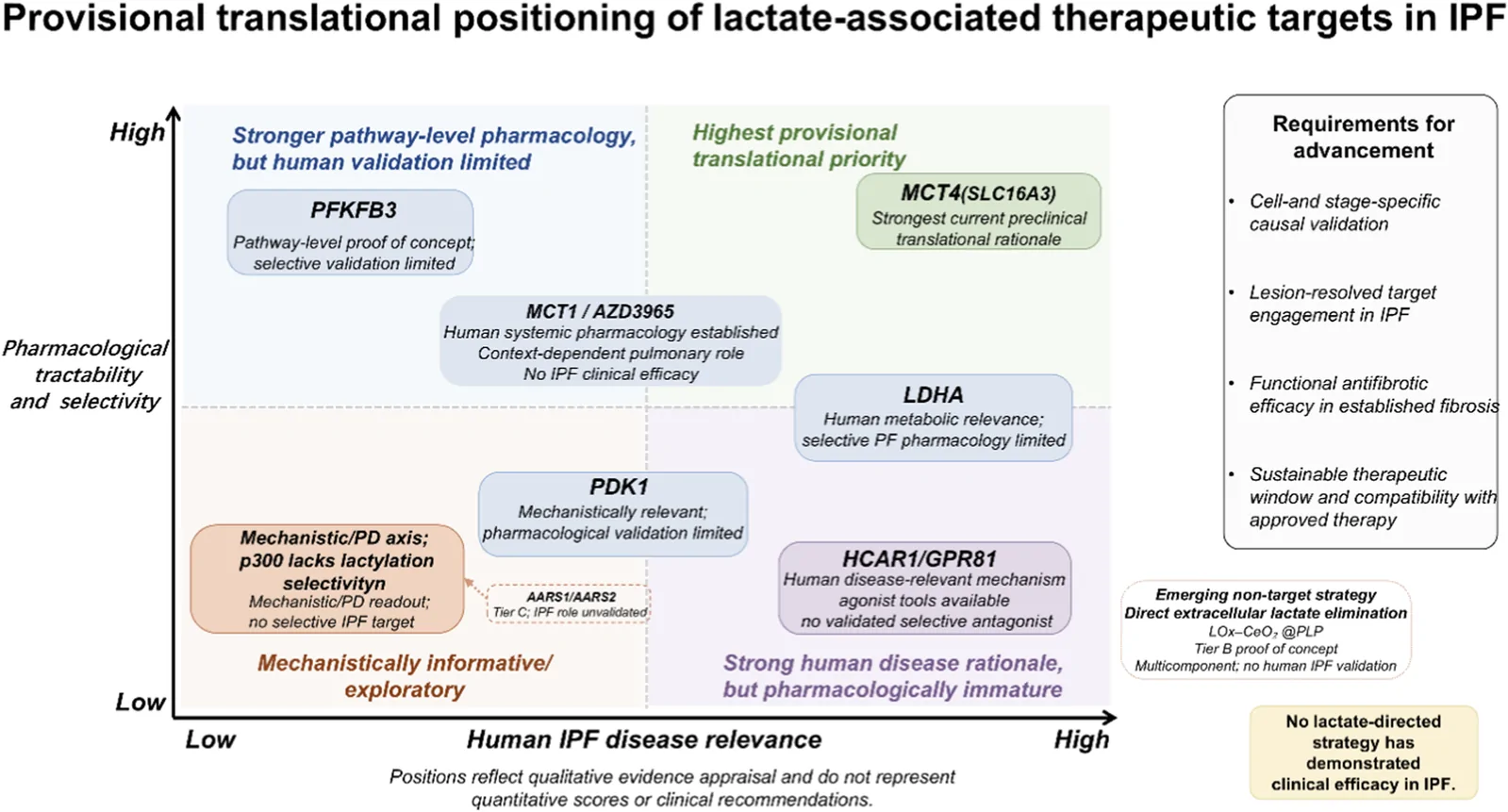
Provisional translational positioning of lactate-associated therapeutic targets in IPF. Candidate targets are positioned according to two complementary dimensions: direct human IPF disease relevance and current pharmacological tractability/selectivity. Fibroblast-associated MCT4 is positioned as having the most developed preclinical evidence package among the lactate-associated targets reviewed. This positioning does not imply clinical validation or a quantitatively established therapeutic ranking. Its provisional position remains contingent on independent confirmation of compound selectivity, pharmacokinetics, safety, therapeutic-stage efficacy, and compatibility with contemporary antifibrotic therapy. PFKFB3 has comparatively strong pathway-level pharmacological proof of concept, but the available interventions do not establish selective PFKFB3-dependent antifibrotic efficacy. LDHA and PDK1 remain mechanistically relevant but pharmacologically less mature. HCAR1/GPR81 has direct disease-relevant mechanistic support in patient-derived stromal populations but remains limited by insufficiently validated selective pharmacological tools. MCT1 is highly context dependent and is not currently prioritized for generalized inhibition. Histone lactylation is better positioned as a mechanistic axis and candidate pharmacodynamic readout than as a stand-alone therapeutic target; p300 lacks lactylation-specific selectivity, and AARS1/AARS2 remain Tier C target-discovery candidates without direct validation in IPF. Positions represent qualitative evidence appraisal rather than numerical scores or clinical recommendations. No lactate-directed strategy has demonstrated clinical therapeutic efficacy in IPF. MCT1 is positioned separately because human oncology studies of AZD3965 establish systemic pharmacological tractability, whereas the context-dependent function of MCT1 and absence of IPF clinical efficacy limit disease-specific prioritization. Direct extracellular lactate elimination is shown as an emerging non-target strategy rather than a ranked molecular target because current evidence derives from a multicomponent lactate-scavenging platform in experimental fibrosis.

## 7 Discussion: critical evidence gaps and translational roadmap

A central question emerging from the available evidence is whether lactate initiates fibrosis or predominantly amplifies and maintains an already established fibrotic microenvironment. Current studies demonstrate that lactate-associated pathways can modify macrophage transcription, fibroblast activation, HCAR1/GPR81-dependent signaling, extracellular-acidification-dependent TGF-β activation, and epithelial stress (Yan et al., 2024a; Yang et al., 2023; Cui et al., 2021; Sun et al., 2024b). However, most mechanistic studies examine systems in which profibrotic stimulation or established fibrotic remodeling is already present. The available evidence is therefore more consistent with lactate functioning as a context-dependent amplifier or maintenance signal than with a demonstrated uniform initiating role in IPF. A particularly important distinction is whether lactate dependence differs among disease initiation, progression, and maintenance. A pathway that is dispensable during initial injury but required to sustain established fibrotic remodeling could still have substantial therapeutic relevance in clinically manifest IPF.

A second major limitation concerns the distinction between a mechanistically supported directional lactate axis and a proposed closed reciprocal fibroblast–macrophage circuit. The strongest direct mechanistic evidence currently supports fibroblast- or myofibroblast-derived lactate acting on macrophages to modify profibrotic transcriptional and lactylation-associated programs (Cui et al., 2021). The lactate-shuttle framework further provides metabolic plausibility for intercellular lactate exchange, and stable-isotope studies demonstrate that extracellular lactate can contribute carbon to pyruvate and TCA-cycle intermediates in lung fibroblasts. However, this metabolic plausibility should not be equated with proof of a fibroblast-to-macrophage oxidative lactate shuttle. No study has yet demonstrated isotope-resolved transfer of fibroblast-derived lactate into macrophages followed by direct quantification of its oxidation, incorporation into lactylated substrates, and subsequent stromal feedback within the same disease-relevant system. Independent studies demonstrate that macrophages can in turn influence fibroblast metabolism, activation, and matrix production through cytokine- and metabolite-dependent pathways, but these directional observations have not yet been connected within a single closed lactate-dependent circuit. Closing this causal loop will require donor-specific lactate perturbation and tracing, macrophage-specific interrogation of lactate uptake, sensing or downstream reprogramming, and demonstration of a macrophage-dependent stromal feedback endpoint within the same disease-relevant system. Until these requirements are met, the term “bidirectional lactate circuit” should be regarded as a working hypothesis rather than an established IPF mechanism.

A third gap is the limited resolution of lactate dependence in human IPF. Current human studies establish disease-associated alterations in lactate metabolism, HIF-associated signaling, fibroblast and epithelial metabolic states, and spatially restricted stromal–immune populations, but they do not determine whether these features coexist within the same lesions or patients. Lactate abundance, extracellular pH, oxygen tension, MCT4-dependent export, HCAR1/GPR81 signaling, and macrophage lactylation have not been simultaneously resolved within human fibrotic niches. Nor is it known whether lactate dependence is broadly shared across IPF or concentrated within a metabolically defined patient subset. Compartmental resolution is equally important because whole-lung, BALF, extracellular, intracellular, and circulating lactate measurements are not interchangeable and cannot by themselves define the concentration or flux experienced by a specific recipient cell within a fibrotic niche. Future human studies should therefore prioritize reproducible lesion- and patient-level phenotypes rather than assume that a pathway identified in a single experimental model or end-stage explant represents a universal feature of IPF. Reproducibility across independent human samples is ultimately more informative for translation than maximal spatial resolution achieved in a single experimental system.

Cell-type specificity represents an additional boundary of the current lactate-centered model. T cells, NK cells, neutrophils, and ILC2s are all relevant to fibrotic lung biology, but the strength of lactate-specific evidence differs substantially among these populations. Human IPF studies support altered lymphoid and granulocytic states, whereas most direct evidence that lactate modifies their effector function derives from non-IPF inflammatory or tumor contexts. Similarly, the ARG1-dependent myeloid–mesenchymal circuit identified in human IPF highlights neutrophil involvement but is ornithine/proline dependent rather than lactate dependent. These observations support broader immune-cell relevance while reinforcing that pulmonary macrophages currently remain the immune compartment with the most direct pulmonary-fibrosis evidence linking increased lactate availability to defined transcriptional and lactylation-associated outputs.

The final translational challenge is not simply target inhibition but therapeutic selectivity. Lactate production, transport, utilization, and sensing contribute to physiological metabolic adaptation, immune function, epithelial repair, and energy homeostasis, making indiscriminate pathway suppression unlikely to be desirable. Therapeutic selectivity may therefore need to arise at several levels, including molecular target selectivity, preferential dependence of disease-associated cell states, spatial enrichment within fibrotic lesions, and disease-stage-specific vulnerability. Lung-directed delivery may improve the therapeutic index, but this advantage should be demonstrated through increased pulmonary target engagement and reduced systemic exposure rather than assumed from local administration alone. Direct extracellular lactate elimination provides an alternative strategy that avoids relying exclusively on inhibition of lactate production, transport or receptor signaling. However, the current lactate-scavenging CeO_2_@PLP platform combines LOx-mediated lactate degradation, ROS modulation, targeted delivery, and pirfenidone, so the observed antifibrotic effect cannot be attributed uniquely to lactate removal. This approach should therefore be viewed as Tier B proof of concept rather than evidence that extracellular lactate depletion is itself a clinically validated therapeutic mechanism. Therapeutic studies should also prioritize established-fibrosis paradigms, prolonged dosing, and age- or comorbidity-relevant models when evaluating metabolic liabilities and durability of response.

A central pharmacological implication is that target validity and druggability should be evaluated independently: pathway prominence alone does not establish that systemic inhibition will provide a favorable therapeutic index, and conversely, human pharmacological tractability does not establish disease-specific target validity. AZD3965 illustrates this distinction. First-in-human oncology studies demonstrate that systemic MCT1 inhibition is pharmacologically achievable, yet MCT1 has a highly context-dependent role in pulmonary lactate handling and AZD3965 has neither demonstrated antifibrotic efficacy nor been clinically evaluated in IPF. By contrast, fibroblast-associated MCT4 currently has stronger IPF/PF disease relevance but remains supported by preclinical pharmacology. HCAR1/GPR81 presents a different limitation again, with disease-relevant mechanistic evidence but insufficiently mature antagonist pharmacology. A practical translational roadmap should therefore require convergence of three forms of evidence before a lactate-associated target is advanced: cell- and disease-stage-specific causal dependence; lesion-resolved evidence that the target participates in pathological lactate coupling in human IPF; and pharmacological demonstration of target engagement, functional antifibrotic efficacy, and a sustainable therapeutic window in established disease. Targets meeting only one of these criteria may remain biologically informative but should not be considered translationally mature. Conversely, reproducible demonstration of all three would provide a stronger basis for progression toward biomarker-informed early-phase clinical evaluation. Equally important, the lactate-centered model should be reconsidered if cell-specific suppression of pathological lactate production or transport fails to alter recipient-cell programs or fibrosis despite adequate target engagement, or if lesion-resolved studies show no reproducible association between lactate-related activity and fibroblast–macrophage neighborhoods in human IPF.

## 8 Conclusion

Current evidence does not establish lactate as a uniform upstream initiating driver of IPF. Instead, available data are more consistent with a context-dependent amplifier or maintenance role within metabolically reprogrammed fibrotic niches. The strongest mechanistic evidence currently supports a directional fibroblast/myofibroblast-to-macrophage lactate axis, whereas a closed reciprocal lactate-dependent fibroblast–macrophage circuit in human IPF remains unproven. Similarly, although lactate-shuttle biology provides a plausible framework for intercellular lactate exchange and oxidative utilization, direct isotope-resolved transfer of fibroblast-derived lactate into macrophages and its subsequent oxidative fate have not been demonstrated in human IPF. Lactate-associated effects on fibroblast activation, extracellular acidification, TGF-β signaling, and epithelial stress further support a broader role in fibrosis maintenance, but these mechanisms vary in disease relevance and causal resolution.

Among the lactate-associated nodes reviewed, fibroblast-associated MCT4 currently has the most developed preclinical evidence package, whereas lactate-production pathways, HCAR1/GPR81, and lactylation-associated machinery remain at earlier stages of target-specific pharmacological validation. Direct extracellular lactate elimination has also emerged as a distinct proof-of-concept strategy, but current evidence remains Tier B and derives from a multicomponent experimental platform, preventing attribution of the total antifibrotic effect specifically to lactate depletion. Lactate-directed strategies should therefore be considered potential complements to approved antifibrotic therapy. Their clinical development will depend on demonstrating cell- and disease-stage-specific causal dependence, lesion-resolved target engagement in human IPF, functional antifibrotic efficacy in established disease, and a therapeutic window that preserves physiological lactate metabolism. The near-term immunopharmacological opportunity is therefore not indiscriminate suppression of lactate metabolism, but cell-, lesion-, and disease-stage-resolved modulation that can achieve target engagement and antifibrotic benefit while preserving physiological lactate handling and immune function.
